# Nonlinear Rheological Behavior of Polyacrylamide Solutions under Large-Amplitude Oscillatory Shear

**DOI:** 10.1021/acs.macromol.5c02848

**Published:** 2026-03-31

**Authors:** Rishav Agrawal, William N. Sharratt, Robert J. Poole, Esther García-Tuñón

**Affiliations:** † School of Engineering, 4591 University of Liverpool, The Quadrangle, Liverpool L69 3GH, United Kingdom; ‡ Materials Innovation Factory, University of Liverpool, 51 Oxford Street, Liverpool L7 3NY, United Kingdom

## Abstract

Understanding how polymer concentration and network structure influence nonlinear rheological response remains a fundamental challenge in polymer physics. Here, we present a comprehensive investigation of the rheology of aqueous polyacrylamide (PAAm) solutions with molecular weight *M*_*w*_ ≈ 5 × 10^6^ g/mol, across a wide concentration range (0.1–12.5 wt %), normalized by the entanglement concentration (*c*_*w*_/*c*_*e*_ = 0.15–19.23). Using steady shear, small-amplitude oscillatory shear (SAOS), and large-amplitude oscillatory shear (LAOS) measurements, we map the evolution from viscous-dominated to elastic-dominated behavior with increasing concentration, accompanied by progressively stronger shear thinning behavior. Nonlinear analyses are performed using Fourier transform (FT) rheology, intrinsic nonlinearity (^3^ *Q*_0_), energy dissipation ratio (ϕ), and the sequence of physical processes (SPP) framework. At low *c*_*w*_/*c*_*e*_, solutions show viscous-dominated behavior with high ϕ values even in the linear regime, whereas higher *c*_*w*_/*c*_*e*_ displays an increasingly elastic response. Beyond the moduli crossover, all samples converge to a plastic-like dissipation plateau (ϕ ≈ 0.85), independent of concentration. SPP analysis reveals a robust intracycle sequence: stiffening, thickening, relaxation, and recoil, whose location and extent depend on both *c*_*w*_/*c*_*e*_ and oscillation frequency. We combine these bulk measures with rheomicroscopy, which uncovers striking differences in flow-induced microstructural behavior. At low concentrations, the flow field remains homogeneous even at high strain. In contrast, highly entangled samples (*c*_*w*_/*c*_*e*_ ≳ 15) show structural disruption, banding, and apparent fracture. These findings highlight that bulk rheology can mask localized instabilities, reinforcing the value of direct imaging approaches. Overall, this study demonstrates the strength of combining normalized concentration scaling with advanced LAOS-based tools (ϕ, ^3^ *Q*_0_, SPP) and imaging to reveal the rich nonlinear rheological behavior of polymer solutions. These insights have implications for soft material design and formulation across diverse applications in printing, flow processing, and biomedical gels.

## Graphical abstract

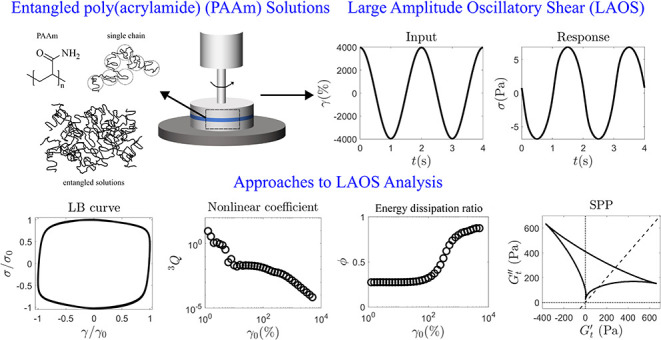

## 1 Introduction

Polymeric materials have become ubiquitous in our everyday life, with their widespread usage ranging from industrial to biological applications as well as in various day-to-day items such as clothes, paints and personal hygienic products. Given their diverse applications, understanding the structure–property relationships of polymeric materials is highly desirable to optimize their functional performance. Rheological measurements are commonplace to give insight into the linear viscoelastic and flow behavior of polymeric materials, typically probed using small amplitude oscillatory shear (SAOS) tests and steady shear deformation tests, respectively.

In recent decades, large amplitude oscillatory shear (LAOS) tests have drawn great interest to characterize polymeric materials^1,2^ (e.g., polymer solutions, melts and nanocomposites),^3–6^ as they better resemble industrial processing operations of these materials, whereby they typically undergo large amplitude deformations. LAOS tests typically probe regions in which the extracted material storage (*G*′) and loss (*G*″) moduli cannot be considered constant and become a function of the strain amplitude. Linear viscoelasticity alone is insufficient to describe and understand nonlinearities arising during LAOS experiments. Consequently there has been substantial concerted efforts to develop new analytical techniques to interpret LAOS data, e.g., Fourier and Chebyshev expansion based techniques, energy dissipation ratio (ϕ), and the Sequence of Physical Processes (SPP) framework discussed here. Lissajous-Bowditch (LB) curves provide visual information on the stress responses to oscillatory shearing.^2,7^ LB curves can be presented as “elastic” representations, plotting the instantaneous stress σ­(*t*) versus instantaneous strain γ­(*t*). The onset of nonlinearity is identified by any distortion to the ellipsoidal pattern of the elastic LB plots.

These curves can be displayed as a function of strain amplitude and applied angular frequency in a Pipkin diagram,^8^ allowing the visualization of the material’s nonlinear response across a broad parameter space. Pipkin space also can be treated as a trajectory space, where different nonlinearities emerge depending on how the system is driven through the γ_0_–ω space (forming the basis for the sequence of physical process or SPP framework discussed in Section 3.7 ^9^). Another approach taken involves Fourier series (FT rheology)^10–12^ and Chebyshev expansions of the stress response.^13^ The interpretation of LAOS data using combined Fourier—Chebyshev expansions (commonly termed FTC rheology) have been widely implemented to understand polymeric systems.^2^ Based on FT rheology, Hyun and Wilhelm^14^ proposed an intrinsic nonlinearity parameter, ^3^ *Q* = (*I*_3/1_)/γ_0_^2^ (where *I*_3/1_ = *I*_3_/*I*_1_, and *I*_1_ and *I*_3_ are the intensities of the first and third harmonics of the stress response, and γ_0_ is the strain amplitude), which has been widely used in probing microstructure evolution of polymeric systems during rheological measurements.^15–17^ Most of the previous studies on polymeric systems have employed a combination of LB curves, ^3^ *Q* and FT rheology (w/o Chebyshev expansions) to investigate nonlinearities arising during LAOS tests.^14,15,18–20^

The energy dissipation ratio (ϕ),^21^ quantifies the energy dissipated in a single oscillation cycle compared to the energy that would be dissipated in a perfect plastic response. This “scalar” measure can be applied to any measured LAOS response and is “well-behaved”, since the strain amplitude and maximum stress are always well-defined and easily determined. Recently, this metric has been applied to study nanocellulose suspensions^22^ and thermoresponsive hydrogels,^23^ and has provided new insights into the energy dissipation mechanisms during LAOS tests. An alternative derivative-based technique, the sequence of physical processes (SPP),^24,25^ has been increasingly applied as a method to analyze LAOS responses. Here, Cole–Cole plots (transient moduli, *G*_*t*_^″^ vs *G*_*t*_^′^ on a linear scale) can provide information about the transient material responses within an oscillation cycle. This technique has been recently implemented to study the nonlinear responses of complex fluids for applications ranging from battery slurries to food products.^9,26–29^ The SPP framework has been applied in more complex rheological experiments, including LAOS experiments simultaneously probed with in situ small-angle neutron scattering, and recovery rheology, where iterative LAOS tests are performed to build transient data sets of recoverable and unrecoverable strains.^30,31^ The demonstrated success of ϕ and the SPP framework in uncovering new insights from LAOS responses of a diverse range of complex fluids has opened new opportunities to extend these techniques to understand the nonlinear behavior of other polymeric materials.

This work focuses on the nonlinear rheology of entangled polyacrylamide (PAAm) solutions. PAAm is a model water-soluble homopolymer for the study of nonlinear flow behavior and widely used industrially. It is utilized as a drag reducing agent in oil recovery,^32,33^ in drug delivery systems,^34–36^ and its most common use as a flocculant.^37,38^ PAAms used in these applications are typically not homopolymers and the term polyacrylamide instead is used to describe a range of copolymers with other (often charged) monomers, e.g., acrylic acid from partial hydrolysis. Reports in the literature often describe PAAms as both neutral polymers and weakly charged polyelectrolytes, owing to differences in preparation method (e.g., polymerization type, type of initiator),^39^ and lead to different interpretations of their flow behavior. We focus on commercially available high-*M*_*w*_ neutral PAAm to systematically explore the evolution of nonlinear rheological behavior with concentration. While PAAm has been extensively studied under steady shear using both bulk rheometry,^39^ creep experiments,^40–42^ and spatially resolved techniques such as Rheo-NMR^43,44^ and optical coherence tomography,^45,46^ comparable insight under large amplitude oscillatory shear (LAOS) remains limited. Prior studies have explored strain-dependent nonlinearities in PAAm solutions,^47^ but a systematic regime-based framework integrating energy dissipation ratio (ϕ) and intracycle measures such as SPP, is lacking. Moreover, despite the widespread evidence of shear banding, wall slip, and flow instabilities under steady flow,^46^ the structural evolution of PAAm under LAOS has not been investigated with comparable depth. Direct visualization of LAOS induced transitions such as banding, microstructural disruption, or fracture remains underexplored, limiting our ability to interpret the nonlinear viscoelastic response in terms of underlying structure.

These issues are addressed by presenting a comprehensive experimental framework, combining steady-shear, oscillatory shear including frequency sweep and strain amplitude sweep, and rheo-microscopy, to characterize nonlinear rheology in aqueous PAAm solutions across a broad concentration range (0.1–12.5 wt %). By covering a wide range of concentrations, distinct microstructural regimes are accessed, from unentangled semidilute solutions to entangled networks, allowing us to interpret the rheological response across applications ranging from low concentration drag-reducing formulations^32,33^ to gel-like systems used in 3D printing.^48^ Moving beyond prior work,^40^ we introduce energy dissipation ratio and SPP analyses to LAOS measurements of high-molecular weight polymer solutions. These advanced descriptors are benchmarked against established tools such as Lissajous–Bowditch (LB) curves and Fourier-transform (FT) rheology, offering new perspectives on the underlying microstructural dynamics. To contextualize the nonlinear response, steady shear and small amplitude oscillatory shear (SAOS) frequency sweeps are performed to assess polymer–solvent interactions, estimate characteristic relaxation times and delineate concentration-dependent rheological regimes.^49^ The LAOS results reveal clear transitions in energy dissipation and intracycle behaviors linked to entanglement onset and transient network formation. Rheo-microscopy provides direct evidence of flow heterogeneities within the sample under LAOS tests, highlighting limitations of bulk rheological results in uncovering local flow behaviors. This integrated approach provides new mechanistic insight and experimental benchmarks for understanding nonlinear viscoelasticity in water-soluble polymers. A schematic overview of the methodology and analysis techniques used for LAOS tests is shown in Figure 1.

**Figure 1:**
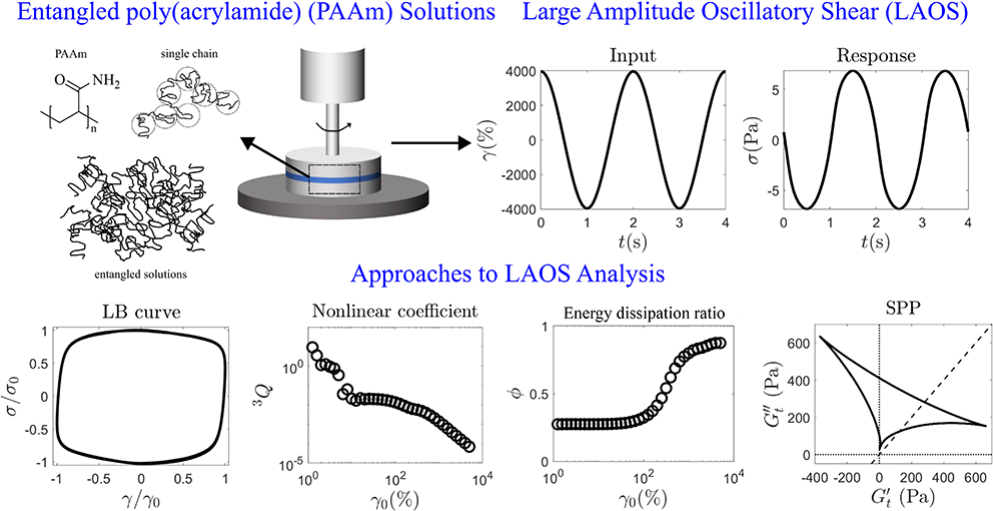
(a) Schematic overview of the methodology and analysis techniques used for LAOS tests in this work. Experiments are performed on a range of concentration of PAAm solutions in a parallel plate geometry. Responses are interpreted in terms of Lissajou-Bowditch (LB) curves, nonlinearity parameter ^3^ *Q*, energy dissipation ratio ϕ and the sequence of physical processes (SPP).

## 2 Materials and Methods

### 2.1 Sample Preparation

Nonionic PAAm with *M*_*w*_ ∼ 4.76 × 10^6^ g/mol and *M*_*w*_/*M*_*n*_ ∼ 2.67, measured by gel permeation chromatography (Figure S1) (nominal *M*_*w*_ ∼ 5 × 10^6^–6 × 10^6^ g/mol—denoted as 5 M PAAm) was purchased from Sigma Aldrich (SKU92560, CAS Number: 9003–05–8). Ten concentrations of aqueous PAAm solutions were prepared: 0.1, 0.25, 0.5, 1, 1.75, 2.5, 5, 7.5, 10, and 12.5 wt%. To prepare the solutions, PAAm powder was initially added to distilled water (pH ≈ 7) and left undisturbed for one day to absorb water. Subsequently, the mixture was gently stirred at approximately 100 rpm for 2–3 days (depending on concentration) at ambient temperature (22 °C) using a magnetic stirrer. For higher concentrations, PAAm powder was added gradually in batches to prevent lump formation, which was found to hinder complete dissolution. Solutions were mixed in airtight cylindrical plastic bottles to minimize evaporation and then rested for approximately 12 h before rheological measurements. Solution homogeneity was checked by sampling from multiple locations (at least three times) within each prepared solution.

The overlap concentration (c*) for PAAm solutions can be estimated either from the intrinsic viscosity [η] via c* = 0.77/[η][Bibr ref49] or from the radius of gyration *R*_*g*_ using c* = (3*M*_*w*_)/(4π*N*_A_ *R*_*g*_^3^),^50,51^ where *N*_A_ is the Avogadro’s number. Using data from François et al.,^52^ with *R*_*g*_ = 0.0749 *M*_*w*_^0.64^ Å and [η] = 0.00933 *M*_*w*_^0.75^ cm^3^/g, and using a nominal *M*_*w*_ (5 M), c* is estimated to be approximately 0.065 wt%. For flexible neutral polymers in good solvents, the entanglement concentration is typically approximated as *c*_*e*_ ∼ 10c*,^51^ giving *c*_*e*_ ≈ 0.65 wt%. It is important to emphasize that, in contrast to other studies,^40,45–47,53^ the PAAm used here is treated as a neutral polymer, as supported by steady shear rheology results (Section 3.1). Previous studies have often used PAAm samples containing significant fractions (∼40 mol%) of acrylic acid monomers, which impart polyelectrolyte character as evidenced in their rheological scaling behavior. Using scaling theories for flexible polyelectrolytes,^41,42^ the entanglement concentration for such copolymeric PAAm samples has been estimated around 0.65 wt%,^46^ which coincidentally aligns with our estimation for neutral PAAm. However, caution is warranted, as some prior approximations^54,55^ have extrapolated c* and *R*_*g*_ values from lower molecular weight systems (e.g., poly­(ethylene oxide), *M*_*w*_ ∼ 1 M) via light scattering measurements, and the propagation of these assumptions may lead to inaccuracies for higher molecular weight PAAm.

For the present work, *c*_*e*_ = 0.65 wt% is considered as the nominal entanglement concentration for the PAAm. Therefore, the concentrations (*c*_*w*_/*c*_*e*_) varies between 0.15 and 19.23 (shown in Table 1). Given these estimates, the lower concentrations (0.1–0.5 wt%; corresponding to *c*_*w*_/*c*_*e*_ = 0.15–0.77) primarily span the semidilute unentangled regime, while concentrations above 1 wt% (*c*_*w*_/*c*_*e*_ = 1.54) enter the semidilute entangled and concentrated regimes.

**Table 1:** Rheological Parameters for Aqueous PAAm Solutions across a Range of Concentrations^a^

*c*_*w*_ (wt %)	*c*_*w*_/*c*_*e*_	η_0_ (Pa·s)	λ_CY_ (s)	*n*	*a*	ω*_c_* (rad/s)	λ (s)	*G*′ = *G*″ (Pa)	*De*_0.1_	*De*_0.5_	*De*_2.5_
0.10	0.15										
0.25	0.38										
0.50	0.77	0.023	0.04	0.725	0.97						
1.00	1.54	0.133	0.07	0.577	1.01	99.6	0.01	2.29	0.006	0.03	0.16
1.75	2.69	2.07	0.60	0.438	0.73	25.0	0.04	5.80	0.02	0.12	0.61
2.50	3.85	22.1	3.03	0.357	0.69	3.96	0.25	9.02	0.16	0.79	3.96
5.00	7.69	708	17.8	0.281	0.92	0.40	2.52	26.9	1.58	7.91	39.6
7.50	11.54	2739	19.0	0.227	1.36	0.25	4.00	66.3	2.51	12.6	62.8
10.00	15.38	1.06 × 10^4^	26.4	0.14	1.19	0.099	10.1	159	6.34	31.7	159
12.50	19.23	1.55 × 10^4^	15.4	0.11	1.72	0.039	25.2	289	15.8	79.1	395

a The table includes Carreau–Yasuda (CY) model fit parameters from steady shear viscosity data (η_0_, λ_CY_, *n*, and *a*); crossover angular frequencies (ω_*c*_), relaxation times (λ), and crossover moduli (*G*′ = *G*″) from small amplitude oscillatory shear (SAOS); and Deborah numbers (*De* = λω) computed at three oscillation frequencies (*f* = 0.1, 0.5, and 2.5 Hz) corresponding to those used in strain amplitude sweep (LAOS) tests.

### 2.2 Rheological Characterization

Rheological measurements were carried out using a strain-controlled ARES G2 rheometer (TA Instruments). Stainless steel, sandblasted parallel plates with a diameter (2*R*) of 40 mm and a fixed geometry gap of 1 mm were used. A solvent trap was employed to minimize solvent evaporation, and the temperature was maintained at 22 °C using a Peltier plate. Steady shear tests were conducted using shear-rate-controlled flow sweeps from 0.005 s^–1^ to 1000 s^–1^ for concentrations *c*_*w*_ ≥ 2.5 wt%, with higher minimum shear rates applied for lower concentrations to ensure reliable measurements. A steady-state sensing protocol with a maximum equilibration time of 20s and a sampling period of 2s per shear rate was employed. At the lowest shear rates, this duration may be shorter than 1/γ̇, and the corresponding data should be interpreted with caution. An additional measurement for the highest concentration (*c*_*w*_/*c*_*e*_ = 19.23) using an extended sensing time of 60 s is provided in the Figure S4 and shows the same viscosity trends, with increased scatter only at the lowest shear rates. Small amplitude oscillatory shear (SAOS) measurements were performed as frequency sweeps (from high to low frequency), with the strain amplitude kept within the linear viscoelastic regime (LVR) for each concentration. The frequency range was selected to ensure that the moduli crossover point could be captured for all samples. SAOS measurements were conducted across concentrations ranging from *c*_*w*_/*c*_*e*_ = 0.77 to 19.23 (lower concentrations data were too close to the resolution of the instrument).

The nonlinear rheological behaviors were investigated using oscillatory strain amplitude sweep tests. In this test, an oscillatory input strain was applied and the resultant output stress was measured for every prescribed strain amplitude (Figure 1). The strain amplitude values (γ_0_) ranging between 1% and 5000% was used in order to investigate the structure deformation from small amplitude oscillatory shear (SAOS) to LAOS. The effect of oscillation frequency (*f*) on the nonlinear response is studied at different *f* = 0.1 Hz, 0.5 Hz and 2.5 Hz, corresponding to angular frequency (ω) = 0.628 rad/s, 3.14 rad/s and 15.7 rad/s, respectively. The ARES G2 rheometer and the TRIOS software can provide the raw transient strain/stress waveforms during a strain amplitude sweep. The transient data were collected for 3 cycles of oscillation for every strain amplitude at a frequency of 1024 points/cycle. Although collecting a larger number of cycles is recommended for high-precision FT-rheology aimed at resolving weak higher harmonics,^56^ three cycles were sufficient here to extract robust LAOS metrics based on waveform and harmonic ratio analyses, consistent with established LAOS analysis frameworks.^13^ Both steady and oscillatory measurements were repeated twice to confirm the repeatability of the results.

Additional rheomicroscopy experiments were conducted for selected PAAm concentrations (*c*_*w*_/*c*_*e*_ = 3.85 and 11.54) following the same setup described in previous work on Pluronic F127 systems.^57^ A stress-controlled Anton Paar MCR702 TwinDrive rheometer, operated in a mode where the bottom plate was driven while the top plate remained stationary, was utilized. Stainless steel, sandblasted 40 mm diameter parallel plates were used with a fixed gap of 1.0 mm. Strain amplitude sweep tests, ranging from γ_0_ = 1% to 565%, were conducted at a fixed oscillation frequency of 0.5 Hz. A custom input waveform was generated externally and loaded via the value list feature in RheoCompass to control nine oscillation cycles per strain amplitude. Instantaneous stress and strain data were recorded every 0.012 s, and the final cycle was used for analysis. Fluorescent polyethylene microspheres of diameter 53–63 μm, purchased from Cospheric (US), were used as tracers. Local structural evolution was monitored using fluorescence microscopy during strain amplitude sweeps. Images were captured at 15.19 fps using a 5× objective and a Basler acA1920–155uc camera with a Sony IMX174 CMOS sensor, yielding a field of view of approximately 1 mm × 1 mm near the edge of the rheometer gap. Rheological and optical data sets were synchronized using MATLAB using inhouse code. Representative fluorescence images were selected from near the peak strain of the final oscillation cycle to illustrate strain-induced structural transitions and flow heterogeneities. Particle tracking and diplacament profiles analysis were not carried out because of the large displacements of the tracer particles (tracers leaving the field of view transiently) just after γ_0_ ≈ 20%. The samples were still in the LVR and no flow instabilties were detected at these amplitudes.

For very soft or low viscosity materials, the minimum transducer torque of the rheometer can be a limitation while carrying out rheological measurements.^58,59^ In these cases, the output stress data can be near the low torque limits of the rheometer and therefore, results can be unreliable. In this study, the effect of low torque limits might be significant at low PAAm concentrations, and is quantified here. For the parallel-plate geometry the minimum measureable stress (σ_min_) is given by, σ_min_ = 2*T*_min_/(π*R* ^3^). Here *T*_min,osc_ = 0.05 μN m and *T*_min,ss_ = 0.1 μN m are the minimum transducer torques in oscillation and steady shear, respectively, as specified by the manufacturer (TA Instruments, ARES G2). This provides minimum measurable stresses in oscillation and steady shear as σ_min,osc_ ≈ 0.016 Pa and σ_min,ss_ ≈ 0.032 Pa, respectively. It also sets limit on the minimum measurable viscoelastic moduli and shear viscosity, *G*_min_ = σ_min_/γ_0_ and η_min_ = σ_min_/γ̇_0_, respectively. At high shear rates, fluid flows may be affected by secondary flows arising from finite sample inertia in curved geometries. To identify this regime, a Reynolds number based criterion is applied for secondary-flow onset in parallel plate geometry, given by η > *H* ^3^ργ̇/*RRe*_crit_,^60^ with *Re*_crit_ ≈ 4.^61^ Data exceeding this limit were excluded from fitting and analysis.

### 2.3 LAOS Data Analysis

LAOS analyses are carried out using Fourier-Transform (FT) rheology,^1,13^ the trends of energy dissipation ratio (ϕ)^21^ and the sequence of physical processes (SPP).^24,25^ The storage and loss moduli, *G*′ and *G*″, are obtained as the first harmonic components of the stress response from a Fourier analysis. For a sinusoidal strain input γ­(*t*) = γ_0_ sin­(ω*t*), the stress response can be represented by a Fourier series^12,62^

1 $$\sigma \left(t;\omega ,\gamma_{0}\right)=\gamma_{0}\sum_{n,\mathrm{odd}}\left\{G_{n}^{\prime }\left(\omega ,\gamma_{0}\right)\sin n\omega t+G_{n}^{\prime \prime }\left(\omega ,\gamma_{0}\right)\cos n\omega t\right\}$$

The first harmonic moduli *G*_1_^′^, *G*_1_^″^ are often referred to as *G*′, *G*″ in the literature and will be so throughout this paper. Higher harmonics obtained from FT-rheology provide a useful measure of intracycle nonlinearities in LAOS.^13,21^ However, their presence is not a necessary condition for nonlinearity, as LAOS responses may remain predominantly single-harmonic in regimes where the material microstructure remains effectively constant, as demonstrated in quasi-linear LAOS and related theoretical studies.^63,64^ Representative stress spectra are provided in the Figure S8 to illustrate the resolved harmonic content and the practical experimental noise floor. At large harmonic numbers, the spectra approach a strain- and concentration-independent plateau at *I*_*n*/1_ ∼ 10^–5^, confirming that the fundamental and low-order harmonics used in the analysis are well resolved above the noise level. A material’s instantaneous properties over the entire oscillation can be visualized using the elastic (stress vs strain) and viscous (stress vs strain rate) LB projections. The LB curves can be further investigated and quantified using an energy dissipation ratio (ϕ) parameter.^21^ It is a ratio of the energy dissipated in a single oscillation cycle (area enclosed in a elastic LB curve, *E*_*d*_) to the energy that would be dissipated in a perfect plastic response with equivalent strain amplitude and maximum stress ((*E*_*d*_)_*pp*_)­

2 $$\phi =\frac{E_{d}}{{\left(E_{d}\right)}_{\mathrm{pp}}}$$

An idealized “perfectly plastic” response corresponds to ϕ = 1, while a purely elastic response corresponds to ϕ = 0.^21^ It is important to note that ϕ is an integral measure based on the shape of the elastic LB curves, and does not differentiate between viscous and plastic contributions to energy dissipation during LAOS experiments. As a result, values of 0 < ϕ < 1 may reflect either viscous or plastic energy loss, or a combination of both, within the material.^57^ It is worth noting that ϕ is directly related to the loss modulus through ϕ = π*G*″ γ_0_/(4σ_0_),^21^ highlighting that ϕ reflects the dissipative information also contained in *G*″ and therefore provides an alternative representation of energy dissipation.

The Sequence of physical processes (SPP) technique, developed by Rogers and co-workers,^24,25^ provides quantitative information for all strain, strain rate, and stress points along the LB curve. The transient elastic modulus (*G*_*t*_^′^) quantifies change in stress w.r.t. strain, and transient viscous modulus (*G*_*t*_^″^) quantifies the change in stress w.r.t. strain rate^26,29^

3 $$G_{t}^{\prime }=\frac{\partial \sigma }{\partial \gamma },,G_{t}^{\prime \prime }=\frac{\partial \sigma }{\partial \left(\frac{\mathrm{\gamma ̇}}{\omega }\right)}$$

The SPP framework is utilized to investigate the intracycle responses in the form of [*G*_*t*_^′^, *G*_*t*_^″^] trajectories of the PAAm solutions as they change during an oscillation cycle. The trace of [*G*_*t*_^′^, *G*_*t*_^″^] trajectories is plotted as a Cole–Cole plot with *G*_*t*_^′^ on the *x*-axis and *G*_*t*_^″^ on the *y*-axis.^25–28,65^ The raw waveform data are analyzed using SPP freeware provided by its developers. The experimentally determined noise floor is used to define the cutoff for Fourier filtering (varying between *n* = 9 and 23) in the SPP analysis and the construction of Cole–Cole plots (Figure S9). Harmonics with normalized intensity at or below the noise plateau (*I*_*n*/1_ ≲ 10^–5^) are excluded to avoid noise-dominated contributions, ensuring that the SPP metrics reflect the measured nonlinear response. At low concentrations and small strain amplitudes, the SPP reconstruction is more susceptible to experimental noise because the nonlinear harmonic content is close to the noise floor (Figure S8), resulting in increased scatter. The SPP analysis is therefore more reliable once nonlinear contributions are well resolved above the noise level.

## 3 Results and Discussion

### 3.1 Steady Shear Rheology

At low shear rates, steady shear viscosity η­(γ̇) approaches a concentration-dependent plateau corresponding to the zero-shear viscosity, η_0_ (Figure 2). As the shear rate increases beyond a critical value γ̇_*c*_, η decreases monotonically (except at very low concentrations, *c*_*w*_/*c*_*e*_ = 0.15 and 0.38, where secondary flows due to finite sample inertia arise at γ̇ ≳ 200 s^–1^), indicating shear thinning behavior characteristic of flexible polymer solutions in good solvents.^39^ The viscosity curves were quantitatively analyzed using the four-parameter Carreau–Yasuda (CY) model by fixing η_∞_ (infinite-shear viscosity) to η_∞_^fix^ = 0.001 Pa s (solvent viscosity), and fitting only the remaining parameters^66^

4 $$\eta_{\mathrm{CY}}\left(\mathrm{\gamma ̇}\right)=\eta_{\infty }^{\mathrm{fix}}+\left(\eta_{0}-\eta_{\infty }^{\mathrm{fix}}\right){\left[1+{\left(\lambda_{\mathrm{CY}}\mathrm{\gamma ̇}\right)}^{a}\right]}^{\left(n-1\right)/a}$$

where η_0_ is the zero-shear viscosity; λ_CY_ is the characteristic time for the onset of shear thinning; *n* is the power-law index at high shear rates; and *a* controls the breadth of the transition between Newtonian and shear-thinning regimes. Fitting was performed by minimizing the normalized squared deviation, $\sum {\left(1-\frac{\eta_{a}}{\eta_{\mathrm{CY}}}\right)}^{2}$ , following established methods for polymer solutions.^66,67^ Here, η_*a*_ is the experimentally measured viscosity and η_CY_ is the viscosity predicted by the Carreau–Yasuda model. At low concentrations, portions of the flow curves affected by instrument noise (at low shear rates due to minimum torque sensitivity) or by inertial effects (at high shear rates) were excluded prior to fitting. The resulting CY fits are overlaid on the experimental data in Figure 2.

**Figure 2:**
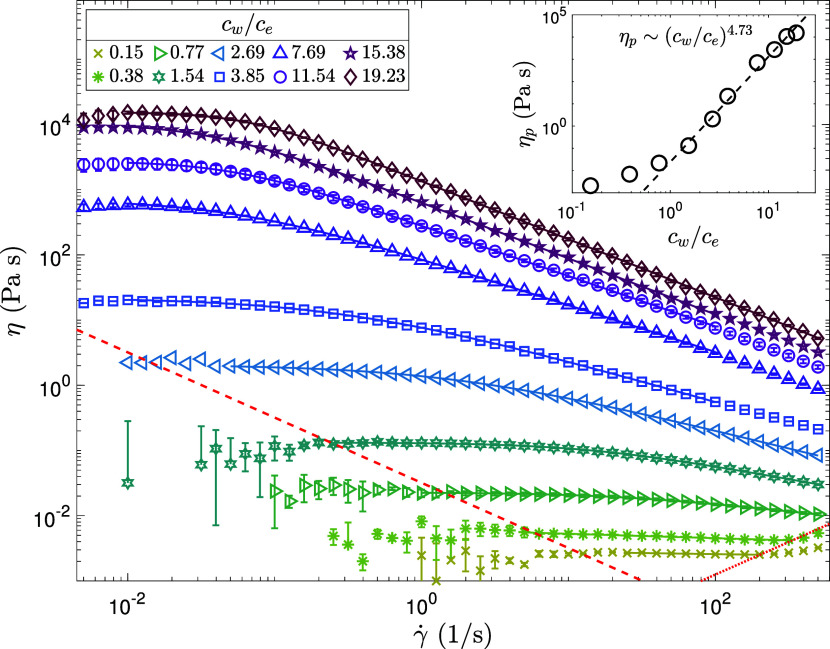
Steady shear viscosity-shear rate profiles for PAAm concentrations ranging from *c*_*w*_/*c*_*e*_ = 0.15–19.23. Solid lines represent fits to the four-parameter Carreau–Yasuda model, obtained by minimizing the normalized squared deviation. The red dashed line denotes the minimum measurable shear stress, σ_min,ss_ ≈ 0.032 Pa, for the ARES G2 rheometer, based on the manufacturer-specified minimum torque *T*_min,ss_ = 0.1 μN·m. The red dotted line at high shear rates indicates the boundary where secondary flows due to finite sample inertia may arise, estimated using the Reynolds number–based criterion, with *Re*_crit_ ≈ 4. The data in this regime are excluded from the CY fitting. Inset on the top right shows the polymeric contribution to the viscosity, η_*p*_ = η_0_ – η_*s*_, as a function of polymer concentration *c*_*w*_/*c*_*e*_, extracted from the Carreau–Yasuda fits. At $c_{w}/c_{e}≳1.54$ , a scaling of η_*p*_ ∼ (c_*w*_/*c*_*e*_)^4.73^ is observed.

To examine the concentration dependence of polymer contributions to viscosity, the polymer concentration to the viscosity, η_*p*_ = η_0_ – η_*s*_ is calculated, where η_*s*_ is the solvent viscosity (DI water in our case). As shown in Figure 2 (inset), at higher concentrations $\left(c_{w}/c_{e}≳1.54\right)$ , a steep scaling of η_*p*_ ∼ (c_*w*_/*c*_*e*_)^4.73^ is observed, indicative of the semidilute entangled regime. This aligns with the theoretical scaling η_*p*_ ∼ *c*_*w*_^3/(3ν–1)^, yielding an exponent of ∼3.9 in good solvents (where ν ≈ 0.588 for good solvents^50^). The onset of this scaling suggests an entanglement concentration *c*_*e*_ near 1 wt% for 5 M PAAm in water, consistent with the empirical rule *c*_*e*_ ∼ 10c* and an estimated critical overlap concentration c* ≈ 0.06 wt%.^51^ The power-law index *n* extracted from CY fits decreases systematically with increasing concentration (Table 1), from *n* ≈ 0.725 in the unentangled regime to *n* ≈ 0.11 in the entangled regime, reflecting increasingly strong shear thinning. The transition breadth parameter *a* varies between 0.5 and 2 across concentrations. These trends from steady shear flow, analyzed via CY modeling and viscosity scaling, provide clear evidence for the concentration-dependent transition from semidilute unentangled to semidilute entangled regimes in PAAm solutions.

### 3.2 Linear Viscoelastic Properties under SAOS test

At lower concentration $\left(c_{w}/c_{e}≲1.54\right)$ , the solutions exhibit viscous-dominated behavior, with cross-over taking place at ω = 99.58 rad/s (Figure 3 and Table 1), indicating the onset of elastic-dominated behavior associated with the formation of a transient entanglement network. At *c*_*w*_/*c*_*e*_ = 2.69, a crossover between storage modulus *G*′ and loss modulus *G*″ is observed at intermediate angular frequency (ω_*c*_ = 25.01 rad/s). At higher concentrations $\left(c_{w}/c_{e}≳11.54\right)$ , a strongly elastic-dominated response is observed, with *G*′ exceeding *G*″ over a broad frequency range (Figure 3 and Table 1).

**Figure 3:**
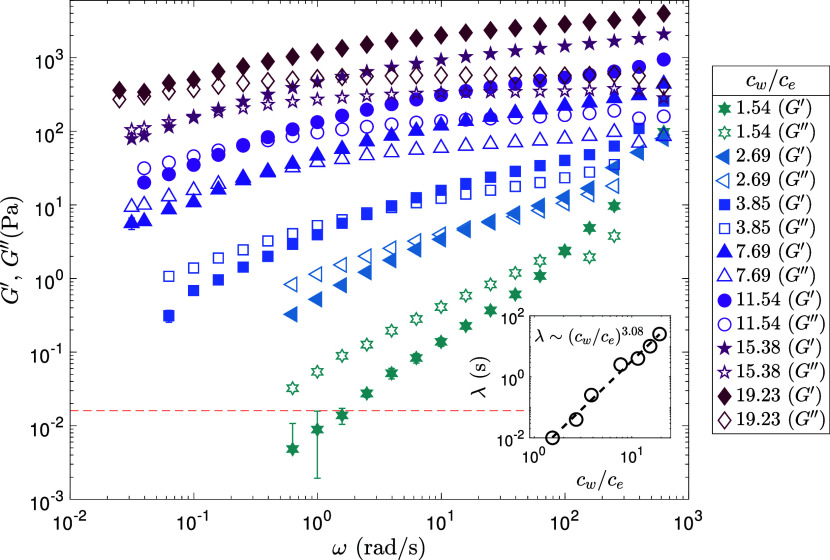
Storage modulus *G*′ and loss modulus *G*″ as functions of angular frequency ω for PAAm aqueous solutions at different concentrations (*c*_*w*_/*c*_*e*_ = 1.54–19.23) at 22 °C. At low concentration $\left(c_{w}/c_{e}≲1.54\right)$ , primarily viscous-dominated behavior (*G*″ > *G*′) is observed across the frequency range, with cross-over taking place at ω = 99.58 rad/s, indicating the onset of entanglement. A crossover between *G*′ and *G*″ is seen at intermediate angular frequency (ω_*c*_ = 25.01 rad/s) for *c*_*w*_/*c*_*e*_ = 2.69. At higher concentrations $\left(c_{w}/c_{e}≳11.54\right)$ , a strongly elastic-dominated response (*G*′ > *G*″) is observed over a broad frequency range. (Inset) Concentration dependence of the characteristic relaxation time λ, extracted from the crossover angular frequency ω_*c*_. A power-law fit yields λ ∼ *c*_*w*_^3.08^, consistent with semidilute entangled dynamics.

The characteristic relaxation time λ for each concentration was estimated from the crossover angular frequency as λ = 1/ω_*c*_, where ω_*c*_ is expressed in radians per second. The extracted crossover frequency and relaxation times are shown in Table 1 and the dependence of λ on *c*_*w*_/*c*_*e*_ is shown in the inset of Figure 3. A power-law fit for (*c*_*w*_/*c*_*e*_) ≥ 1.54 yields λ ∼ (c_*w*_/*c*_*e*_)^3.08^. In semidilute entangled polymer solutions, classical scaling theories predict that the relaxation time scales with concentration as λ ∼ *c*_*w*_^(3ν)/(3ν–1)^, where ν ≈ 0.588 for good solvents,^50^ leading to λ ∼ (c_*w*_/*c*_*e*_)^2.3^. This scaling is characteristic of semidilute entangled flexible polymer solutions in good solvents, where stress relaxation is dominated by reptation dynamics.^50^ The observed steeper scaling exponent of 3.08 in our PAAm solutions suggests that the system is firmly within the semidilute entangled regime, but with stronger concentration dependence than predicted by ideal scaling theory. Such deviations are commonly attributed to factors including increased chain stiffness, concentration-enhanced hydrodynamic screening, or slight deviations from good solvent conditions.^51^

### 3.3 Storage and Loss Moduli, *G*′ and *G*″

At low PAAm concentrations, *c*_*w*_/*c*_*e*_ ≤ 1.54, the moduli show a predominantly viscous behavior with *G*″ > *G*′ at all strains and oscillation frequencies (Figure S3a). A high level of uncertainty in the *G*′ values at low strains and frequencies is observed due to the measured torque being close to the minimum torque limit (Section 2.2).

For *c*_*w*_/*c*_*e*_ = 3.85, the moduli show different behaviors at different oscillation frequencies, where *f* = 0.1, 0.5 and 2.5 Hz correspond to *De* = ωλ = 0.158, 0.791 and 3.957, respectively (Figure 4a). Here, the Deborah number is defined using the relaxation time extracted from linear viscoelastic measurements (Table 1) and is used as a reference frequency parameter. Viscous-dominant behavior is observed at *De* = 0.158 with *G*″ > *G*′ across all strains. At *De* = 0.791, the moduli in the linear viscoelastic region (LVR) (*G*_LVR_^′^, *G*_LVR_^″^) show *G*_LVR_^″^ ≈ *G*_LVR_^′^, indicating a transition from viscous-dominated to elastic-dominated response, and at *De* = 3.957, *G*_LVR_^′^ > *G*_LVR_^″^ is observed. At PAAm concentrations *c*_*w*_/*c*_*e*_ = 7.69 and above, the moduli exhibit behavior typical of strongly elastic networks at all oscillation frequencies, with *G*′ > *G*″ at small strains and a *G*′ = *G*″ crossover at intermediate strains (Figure S3). It is also noted that *G*″ does not show an overshoot at intermediate strain values for any PAAm concentration or frequency (Figure S3). The absence of a *G*″ overshoot is typically associated with Type I (strain thinning) nonlinear behavior,^68^ as opposed to systems exhibiting overshoot behavior, such as Pluronic F127 hydrogels.^23^

**Figure 4:**
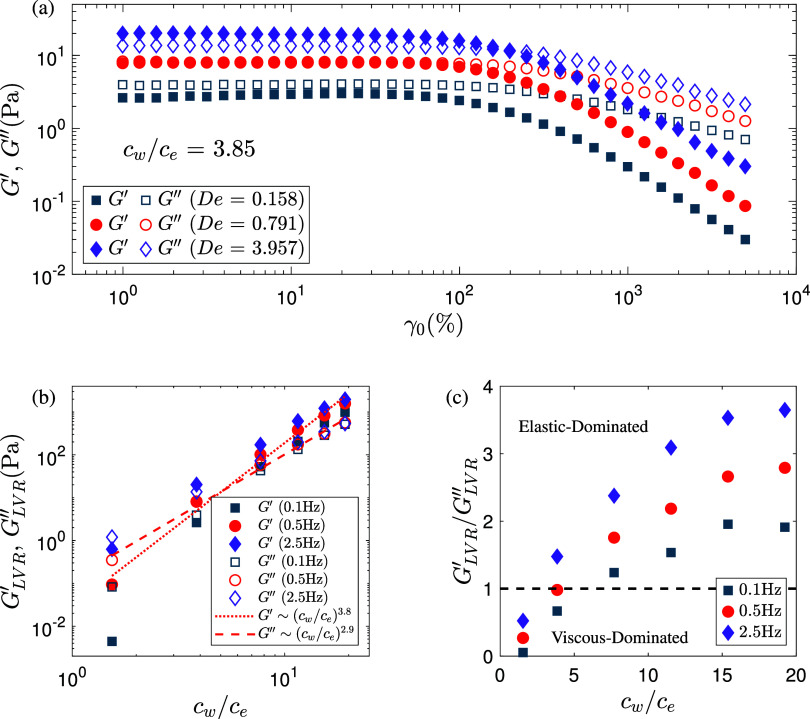
(a) Variation of *G*′ and *G*″ with increasing strain amplitudes at various oscillation frequencies for *c*_*w*_/*c*_*e*_ = 3.85. (b) *G*_LVR_^′^ and *G*_LVR_^″^, and (c) *G*_LVR_^′^/*G*_LVR_^″^ as a function of PAAm concentration for different oscillation frequencies. The red dotted and dashed lines in (b) highlight the scaling, *G*_LVR_^′^ ∼ (c_*w*_/*c*_*e*_)^3.8^ and *G*_LVR_^″^ ∼ (c_*w*_/*c*_*e*_)^2.9^, respectively. The dashed line in (c) represents *G*_LVR_^′^/*G*_LVR_^″^ = 1, used to demarcate elastic-dominated and viscous-dominated behavior.

The storage and loss moduli in the LVR increase with increasing PAAm concentration (Figure 4b), similar to the behavior observed in steady shear (Section 3.1). The effect of oscillation frequency is more pronounced at low concentrations, where *G*_LVR_^′^ and *G*_LVR_^″^ both increase with increasing *f*. This behavior arises because faster oscillations provide less time for polymer chains to relax, resulting in greater elastic energy storage. At higher concentrations, the moduli exhibit weaker dependence on *f*, consistent with behavior typical of dynamically arrested physical gels.^69^ PAAm solutions, while not chemically cross-linked, can form physically entangled and hydrogen-bonded networks, similar to other associative polymers such as poly­(vinyl alcohol).^70,71^ These transient structures can give rise to rheological responses reminiscent of yield stress fluids: solid-like at low strains and fluid-like at large strains.^72^ To probe the existence of a yield stress, a recently proposed ‘Protorheology’ method^73^ is employed, in which a sample is deformed and its ability to retain shape under gravity is monitored over extended times. The highest concentration PAAm solution (*c*_*w*_/*c*_*e*_ = 19.23) was placed on a PTFE substrate and left undisturbed for 2 h (Figure S2). The sample was observed to flow slowly under gravity over this time scale, indicating that any yield stress present is weak and insufficient to prevent deformation under its own weight. This qualitative observation is consistent with the steady shear measurements, which do not exhibit a clear yield-stress plateau at low shear rates even at the highest concentration (Figure S5). Together, these results indicate that the system behaves as a strongly shear-thinning viscoelastic material rather than a material with a well-defined yield stress.

The frequency dependence with increasing concentration becomes less prominent for *G*_LVR_^″^, particularly at the highest concentrations where *G*_LVR_^″^ becomes nearly independent of *f* (Figure 4b). In contrast, *G*_LVR_^′^ retains some mild frequency dependence across all concentrations. The ratio *G*_LVR_^′^/*G*_LVR_^″^ consistently increases with increasing *f* (Figure 4c), demonstrating that higher oscillation frequencies enhance elastic contributions irrespective of concentration. Important to note here that even at a fixed oscillation frequency, the Deborah number (*De* = *λω*) varies with concentration due to the increasing relaxation time λ (Table 1). As a result, changes in viscoelastic response across concentrations cannot be attributed to frequency alone. Instead, the observed trends reflect a growing dominance of slow structural relaxation processes at higher concentrations due to increased chain entanglements and interactions,^50^ which increase *De* and amplify elastic behavior even at the same *f*.

Across steady shear, linear viscoelastic, and strain amplitude sweep measurements, a coherent picture emerges of the microstructural evolution of PAAm solutions with increasing concentration. Steady shear flow behavior reveals two regimes, with a transition from semidilute unentangled to semidilute entangled dynamics at around 1 wt% (*c*_*w*_/*c*_*e*_ = 1.54), as indicated by a change in the η_*p*_ scaling. SAOS measurements corroborate this transition, showing a crossover between *G*′ and *G*″ at around 1.75wt% (*c*_*w*_/*c*_*e*_ = 2.69) and increasingly elastic behavior at higher concentrations. Strain amplitude sweeps reveal that for *c*_*w*_ ≥ 5 wt% (*c*_*w*_/*c*_*e*_ ≥ 7.69), the solutions exhibit dominant elasticity at small strains and crossover at larger deformations. Together, these results demonstrate that PAAm solutions evolve from viscous-dominated fluids to transiently arrested, physically entangled networks displaying elastic-dominated behavior under small deformations. The consistent transition points identified across different rheological protocols reinforce the robustness of this interpretation.

### 3.4 Characterizing Nonlinearities from Analysis of FT-Rheology

FT-rheology provides a quantitative method to determine the onset of nonlinearities in the stress response, marking the end of the linear viscoelastic region (LVR), here denoted by γ_*nl*_. This is determined based on the appearance of the third harmonic in the oscillatory stress response,^74^ defined when the intensity of the third harmonic (*I*_3_) exceeds the defined threshold criterion of 0.1% of the first harmonic (*I*_1_), i.e., *I*_3/1_ > 0.001.^28,75^ Although this criterion is somewhat arbitrary, the γ_*nl*_ values obtained correspond closely to the point where the *I*_3/1_ scaling transitions from *I*_3/1_ ∼ γ_0_^–1^ to *I*_3/1_ ∼ γ_0_^2^ (Figure 5a), a transition commonly associated with the boundary between the small-amplitude oscillatory shear (SAOS) and medium-amplitude oscillatory shear (MAOS) regimes.^2,15^ At lower concentrations (*c*_*w*_/*c*_*e*_ ≤ 3.85), *I*_3/1_ remains above the nonlinearity threshold at *f* = 0.5 Hz, coinciding with a predominantly liquid-like linear viscoelastic response with *G*″ > *G*′ across all strain amplitudes (Figure S3a,b). For *c*_*w*_/*c*_*e*_ ≥ 7.69, the *I*_3/1_ curves are nearly indistinguishable (Figure 5a), suggesting a convergence in nonlinear response across concentrations.

**Figure 5:**
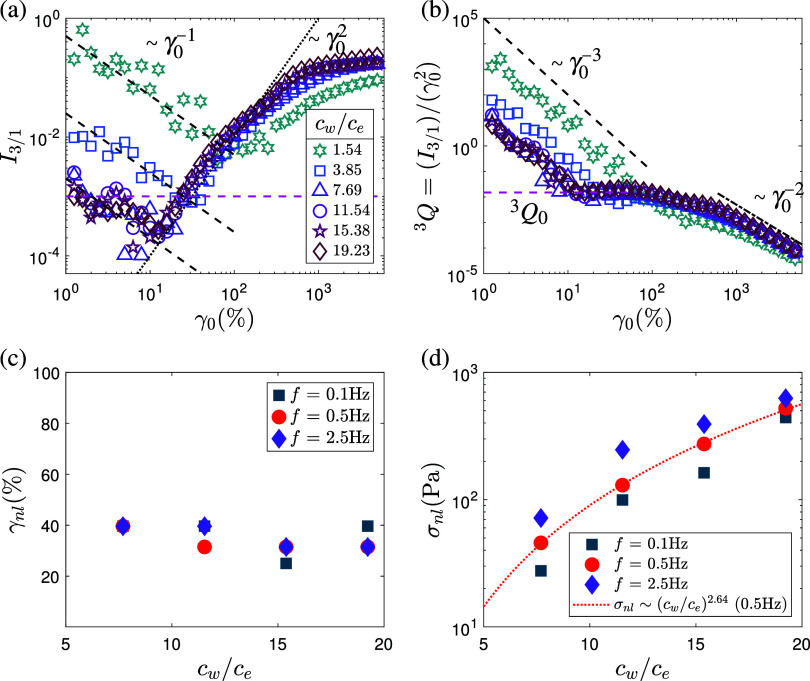
(a) Normalized intensities of the third harmonic (*I*_3/1_) as a function of strain amplitude (γ_0_, %) for different PAAm concentrations at *f* = 0.5 Hz (solid lines). The dashed purple line represents the threshold for the onset of nonlinearity (*I*_3/1_ > 0.001). Black dotted and dashed lines indicate *I*_3/1_ ∼ γ_0_^2^ and *I*_3/1_ ∼ γ_0_^–1^ scaling, respectively. (b) Nonlinear parameter (^3^ *Q*) as a function of γ_0_ for different PAAm concentrations at *f* = 0.5 Hz. Dashed purple lines indicate extrapolation to determine ^3^ *Q*_0_. (c) Characteristic strain γ_*nl*_ and (d) corresponding stress σ_*nl*_ at the onset of nonlinearity, shown as functions of PAAm concentration for different oscillation frequencies. Results for *c*_*w*_/*c*_*e*_ ≤ 3.85 are not shown in (c, d) due to viscous-dominated behavior across all strain amplitudes.

Three distinct regimes of third harmonic contribution can be identified with increasing strain amplitude γ_0_. In Region I (SAOS), *I*_3/1_ decreases as *I*_3/1_ ∼ γ_0_^–1^, indicating that the third harmonic remains below the sensitivity limit of the rheometer.^76,77^ In Region I, *I*_3_ ∼ γ_0_^0^ while *I*_1_ ∼ γ_0_^1^, leading to *I*_3/1_ ∼ γ_0_^–1^. The strain range over which this scaling holds is largest at low concentrations (e.g., γ_0_ ≈ 80% at 1 wt%), and decreases with increasing concentration and entanglement density, reaching γ_0_ ≈ 20% at the highest concentrations. In Region II (MAOS), *I*_3/1_ scales approximately quadratically with γ_0_, *I*_3/1_ ∼ γ_0_^2^ (Figure 5a), consistent with the emergence of intrinsic nonlinearities commonly reported in polymeric and soft-matter systems under oscillatory shear.^19,78^ In this regime, the normalized nonlinear parameter ^3^ *Q* = (*I*_3/1_)/γ_0_^2^ becomes constant, and the intrinsic nonlinearity ^3^ *Q*_0_ is obtained as lim_γ_0_→0_^3^ *Q* (Figure 5b). At larger strains (Region III, LAOS), *I*_3/1_ deviates from the quadratic scaling. The third harmonic contribution follows an approximate ^3^ *Q* ∼ γ_0_^–2^ dependence, which is used here as a diagnostic indicator of pronounced nonlinear response, similar to behaviors observed in mono- and polydisperse homopolymer melts.^15^

Following the approach described by Wilhelm et al.^10–12^ and recent developments by Ewoldt et al.,^13^ the onset of measurable nonlinearity in oscillatory shear is characterized via higher harmonic generation in FT-rheology. Although this transition is sometimes referred to as “yielding” in viscoelastic fluids, it is important to emphasize that our PAAm solutions do not exhibit true yield stress behavior, even at the highest concentrations studied (discussed in Section 3.3). Therefore, the quantities we denote as γ_*nl*_ and σ_*nl*_ correspond to the onset of nonlinear viscoelasticity, not to classical yielding. Within this framework, the onset strain for nonlinearity, γ_*nl*_, remains relatively constant at approximately 40% across all tested concentrations (7.69 ≤ *c*_*w*_/*c*_*e*_ ≤ 19.23) and oscillation frequencies (Figure 5c). In contrast, the corresponding stress at this point, σ_*nl*_, increases markedly with concentration, rising nearly an order of magnitude between *c*_*w*_/*c*_*e*_ = 7.69 and 19.23 (Figure 5d). This trend likely reflects the increasing entanglement density and energy required to drive structural rearrangement under oscillatory deformation. Higher oscillation frequencies also lead to higher σ_*nl*_, consistent with increased chain orientation, stretching, and enhanced intermolecular interactions under faster deformation. Overall, γ_*nl*_ and σ_*nl*_ provide a useful operational measure for comparing the onset of nonlinear viscoelasticity across conditions, but they should not be interpreted as indicators of a static yield point or yield stress material behavior in the classical sense.^79^

### 3.5 Energy Transition Mechanisms Based on the Energy Dissipation Ratio (ϕ)

At low strain amplitudes, the elastic LB curves are nearly elliptical, indicative of predominantly linear elastic behavior (Figure S6). With increasing PAAm concentration, the area enclosed by these curves decreases, reflecting reduced energy dissipation and a more elastic response (Figure S6). A similar trend is observed with increasing oscillation frequency in the linear viscoelastic regime (LVR), consistent with the increasing ratio *G*_LVR_^′^/*G*_LVR_^″^ (Figure 4c). As the strain amplitude exceeds γ_*nl*_, the LB curves begin to distort from their elliptical shape, marking the onset of nonlinear behavior. At very large strains (γ_0_ > 1000%), corresponding to the bulk flow regime, the LB curves across different concentrations converge toward near-square shapes with rounded corners, typical of shear-thinning viscoelastic fluids such as xanthan gum and is consistent with prior observations of Carreau-like shear thinning systems.^21^ This rectangular distortion reflects increasing dominance of nonlinear viscous dissipation during deformation. To complement the elastic LB plots, viscous LB plots (stress vs strain-rate) are also presented in the Figure S7 to further elucidate the time-resolved material response at different concentrations and strain amplitudes.

The evolution of LB curve morphology with increasing concentration bears resemblance to behaviors reported for materials often described as “apparent yield stress fluids”.^21,24^ Such systems, though not truly yielding, can exhibit strong pseudoplasticity or elastoviscoplastic-like responses, where energy is primarily stored elastically at small deformations and dissipated nonlinearly at large ones.^26^ In our case, however, we have shown that PAAm does not possess a true yield stress, even at the highest concentrations. In the LVR, ϕ decreases systematically with concentration; for example, at *f* = 0.5 Hz and *c*_*w*_/*c*_*e*_ = 19.23, ϕ_LVR_ ≈ 0.25 (Figure 6a,f), indicating a more elastic-like response, where as for *c*_*w*_/*c*_*e*_ = 3.85, ϕ_LVR_ ≈ 0.54. Due to significant noise, ϕ values at *c*_*w*_/*c*_*e*_ ≤ 1.54 are considered unreliable and are not shown.

**Figure 6:**
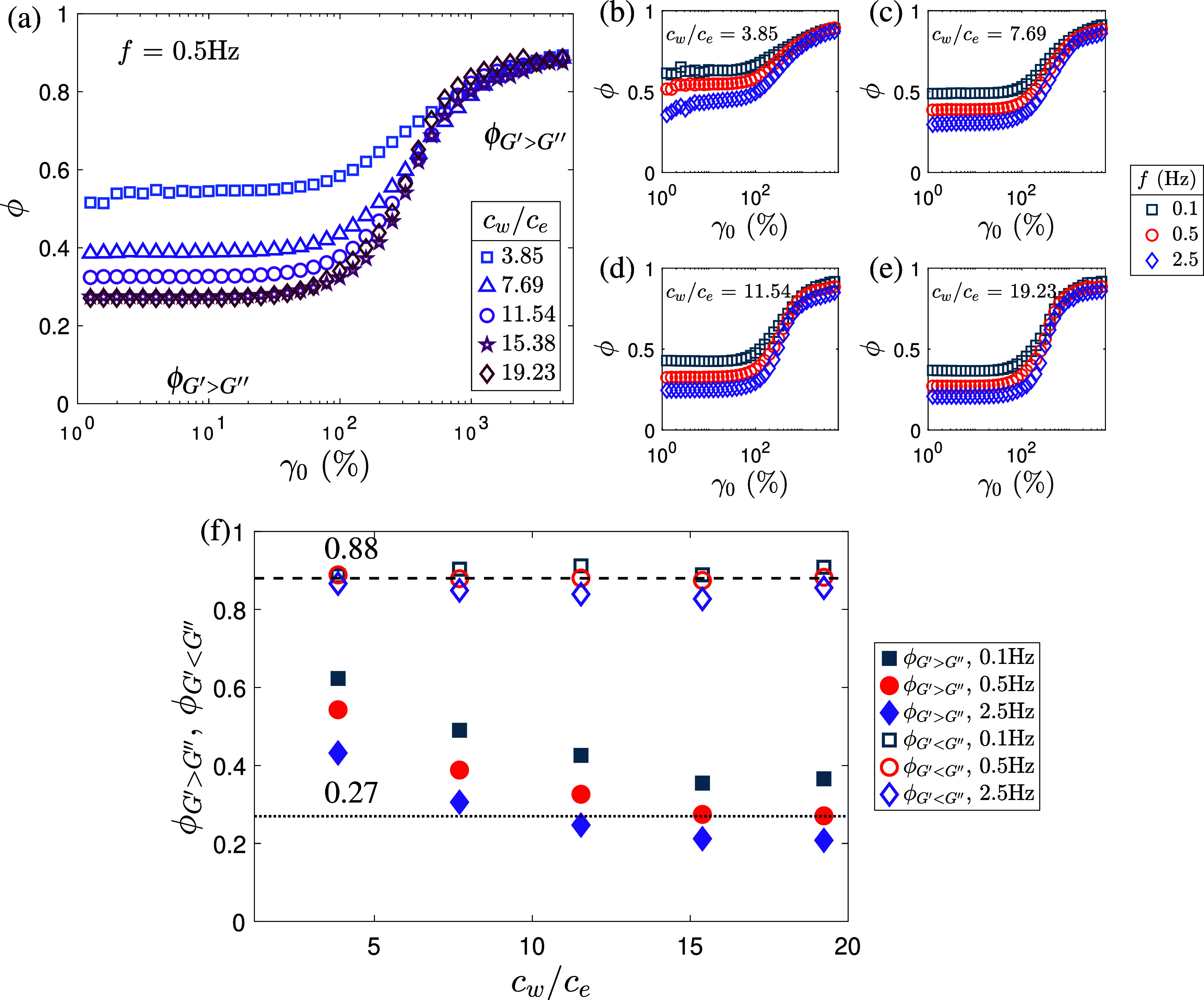
(a) Variation of the energy dissipation ratio (ϕ) with strain amplitude (γ_0_) for different PAAm solutions at oscillation frequency, *f* = 0.5 Hz. Effect of *f* on ϕ for *c*_*w*_/*c*_*e*_ = (b) 3.85, (c) 7.69, (d) 11.54 and (e) 19.23. (f) ϕ_LVR_ and ϕ_flow_ as a function of PAAm concentrations for different oscillation frequencies. ϕ_*G*′>*G*″_ and ϕ_*G*′<*G*″_ are calculated at γ_0_ = 8% and 3960%, respectively (shown in (a)). The horizontal dotted and dashed lines in (f) indicate constant values of 0.27 and 0.88, respectively. The ϕ data for *c*_*w*_/*c*_*e*_ ≤ 1.54 is not shown due to very high level of noise in the LB curves as seen in Figure S6.

Beyond the LVR, ϕ increases markedly with strain amplitude for all concentrations, transitioning toward values ϕ ∼ 0.8–0.9 at large deformations. The *c*_*w*_/*c*_*e*_ = 19.23 PAAm solution shows the steepest increase, transitioning from ϕ ≈ 0.25 (elastic-like) to ϕ ≈ 0.9 (plastic-like) as strain increases. In contrast, lower concentration solutions (e.g., *c*_*w*_/*c*_*e*_ = 3.85) exhibit a more gradual increase in ϕ (Figure 6a). At very large strains (γ_0_ ≳ 1000%), ϕ becomes similar across all concentrations and frequencies, converging to an asymptotic value around 0.8–0.9. This is consistent with FT-rheology observations in other pseudoplastic materials, such as xanthan gum solutions, where ϕ asymptotes to approximately 0.87.^21^ The incomplete approach to ϕ = 1 indicates persistent viscous dissipation, characteristic of pseudoplastic rather than perfectly plastic yielding behavior. Phenomenologically, this behavior can be explained as follows; at small deformations, polymer chains relax rapidly, and the entangled network remains intact, resulting in elastic recovery. Increasing oscillation frequency enhances the elastic-like response, as *De* increases, due to insufficient time for chain relaxation between cycles. It is speculated that hydrogen bonding between PAAm chains may contribute significantly to maintaining microstructural integrity under small deformations. At large strains, chain disentanglement, stretch, and orientation dominate the mechanical response, facilitating plastic or viscous (or both) energy dissipation. In contrast to conventional hydrophobic polymer melts, the aqueous PAAm solutions investigated here display pronounced nonlinearities and flow instabilities (at high concentrations, Figure 7), likely due to both entanglements and physical associations (e.g., hydrogen bonds).

**Figure 7:**
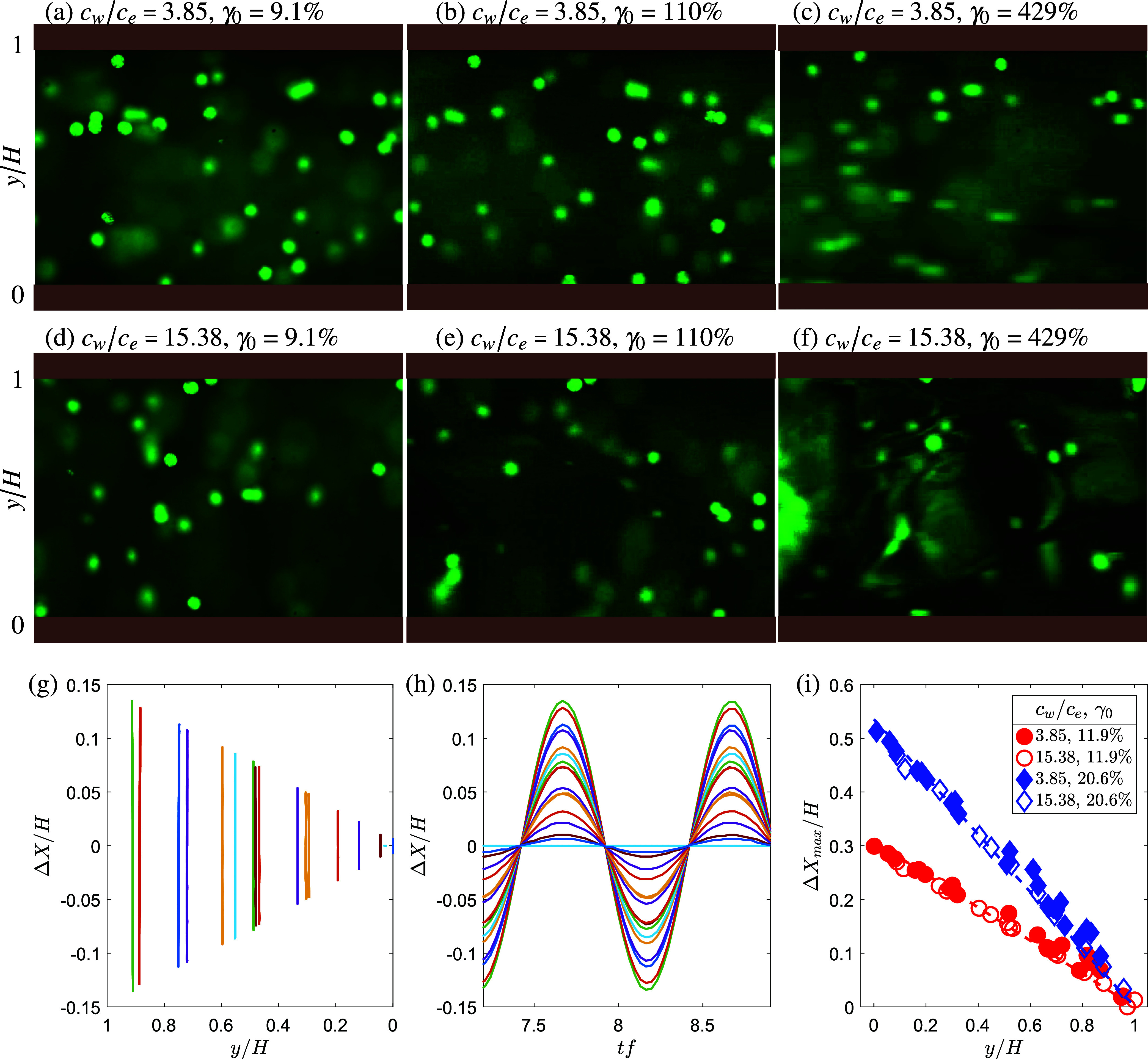
Rheo-microscopy images and displacement analyses of PAAm solutions during LAOS at *f* = 0.5 Hz. Top row (a–c): *c*_*w*_/*c*_*e*_ = 3.85; bottom row (d–f): *c*_*w*_/*c*_*e*_ = 15.38. Strain amplitude increases from left to right (γ_0_ = 9.1%, 110%, 429%). At low strain amplitudes (LVR), the flow remains homogeneous, while increasing strain and concentration lead to pronounced microstructural distortion and spatial heterogeneity. Panels (g, h) show projections of the time-dependent tracer displacement trajectories (Δ*X* = *X* – ⟨*X*⟩, where ⟨*X*⟩ is the average displacament) across the gap in the LVR for *c*_*w*_/*c*_*e*_ = 15.38 at γ_0_ = 11.9%, demonstrating affine deformation. Panel (i) shows the corresponding maximum displacement (Δ*X*_max_ = *X*_max_ – *X*_min_) profiles across the gap at two strain amplitudes, γ_0_ = 11.9% and 20.6% which remain linear in the LVR. At larger γ_0_, quantitative time-resolved displacement analysis is not shown due to tracer particles leaving the field of view.

While the evolution of ϕ provides valuable insight into how the relative importance of energy dissipation changes during deformation, it remains an inherently bulk-averaged quantity. As such, it cannot resolve whether the nonlinear response arises uniformly throughout the sample or is instead driven by localized structural transitions. Previous work on micellar systems and polymer solutions has shown that phenomena such as shear banding, fracture, or microstructural failure often originate from highly localized events that remain invisible to conventional bulk rheological measurements.^46,47,80^ To directly probe these spatial and temporal deformation patterns, we next turn to rheomicroscopy, which provides real-time visualization of structural evolution across the strain amplitude spectrum.

### 3.6 Flow Heterogeneities Observed via Rheomicroscopy

The videos (see Supporting Videos S1 and S2) provide real-time visualization of how the local flow evolves as the material is driven from the linear regime into the nonlinear regime. These observations complement the quantitative bulk rheology and help identify the emergence of spatial heterogeneity, localized deformation, and microstructural failure. At *c*_*w*_/*c*_*e*_ = 3.85 and 15.38, the structure remains largely homogeneous at low strains (γ_0_ ≲ 20%), with uniform contrast (Figure 7a–f). The corresponding maximum displacement profiles extracted at two strain amplitudes remain linear across the gap, consistent with homogeneous affine deformation (Figure 7g–i). At *c*_*w*_/*c*_*e*_ = 3.85, as the strain increases, even at large strains (γ_0_ ≈ 429%), the flow field remains mostly homogeneous, with no abrupt failure (see Supporting Videos S1 and S2). This behavior is consistent with gradual energy dissipation observed in ϕ and the smooth evolution of SPP trajectories (as shown later in Section 3.7).

By contrast, the *c*_*w*_/*c*_*e*_ = 15.38 sample shows pronounced local flow heterogeneities with increasing deformation. Shear bands emerge with increasing deformation eventually giving way to fracture within the sample. At high strain amplitudes (γ_0_ > 100%), the *c*_*w*_/*c*_*e*_ = 15.38 sample shows abrupt contrast loss and smearing, strongly suggestive of fracture-like behavior. These observations also agree with the previously reported banding instabilities in entangled PAAm solutions at large deformations.^47^ Crucially, this failure cannot be inferred from bulk rheological measurements alone. For example, ϕ shows similar asymptotic values at high γ_0_ for both low and high concentrations (Figure 6), suggesting a converging plastic-like regime.^21^ However, rheomicroscopy reveals that the mechanisms leading to this regime differ fundamentally: homogeneous flow at low concentrations versus intense banding and fracture at high concentrations. This raises questions about how to interpret bulk metrics like ϕ or harmonic ratios in isolation, especially in entangled polymer systems where spatial heterogeneities and failure modes are strain- and concentration-dependent. Together, these results highlight the necessity of combining rheometry with direct imaging to capture the nature of nonlinear deformation in complex fluids.

### 3.7 Microstructure Evolution Using the Sequence of Physical Processes (SPP) Framework

The Cole–Cole plots for all solutions exhibit the characteristic deltoid shape i.e., a triangular closed loop (Figures 8 and 9), reflecting the dominance of the third harmonic.^25^ The location, orientation and area of the deltoids provide valuable insights into the sequence of microstructural evolutions the material undergoes during each oscillation cycle. The location of the deltoids provides information regarding the nature of deformation, the orientation reflects the nature of the material’s deformation transition, while the area shows the extent of microstructural rearrangements.^9,24^ Left or right movements in this plot are associated with softening or stiffening, respectively, whereas top or bottom movement are associated with thinning or thickening, respectively. When *G*_*t*_^″^ > *G*_*t*_^′^, a response would be considered predominantly viscous, while *G*_*t*_^″^ < *G*_*t*_^′^ would be considered predominantly elastic response.^25^ To build on the spatially resolved insights from rheomicroscopy and elucidate how microstructural transitions evolve dynamically under deformation, the stress response is analyzed using the sequence of physical processes (SPP) that occur within each oscillation cycle, as captured by the characteristic deltoid shapes in the Cole–Cole plots (Figures 8 and 9).

**Figure 8:**
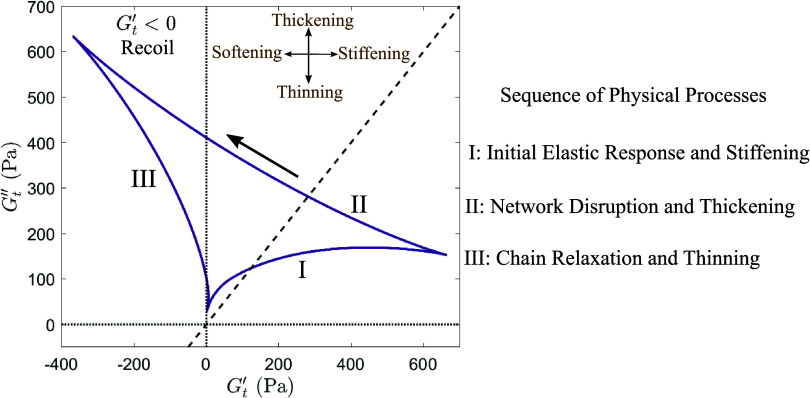
SPP analysis of the intracycle rheological transition within an oscillation cycle at *f* = 0.5 Hz using Cole–Cole plot for PAAm concentration of *c*_*w*_/*c*_*e*_ = 19.23 at γ_0_ = 995%. The dashed line represents *G*_*t*_^′^ = *G*_*t*_^″^ and the vertical and horizontal dotted lines represent *G*_*t*_^′^ = 0 and *G*_*t*_^″^ = 0, respectively.

**Figure 9:**
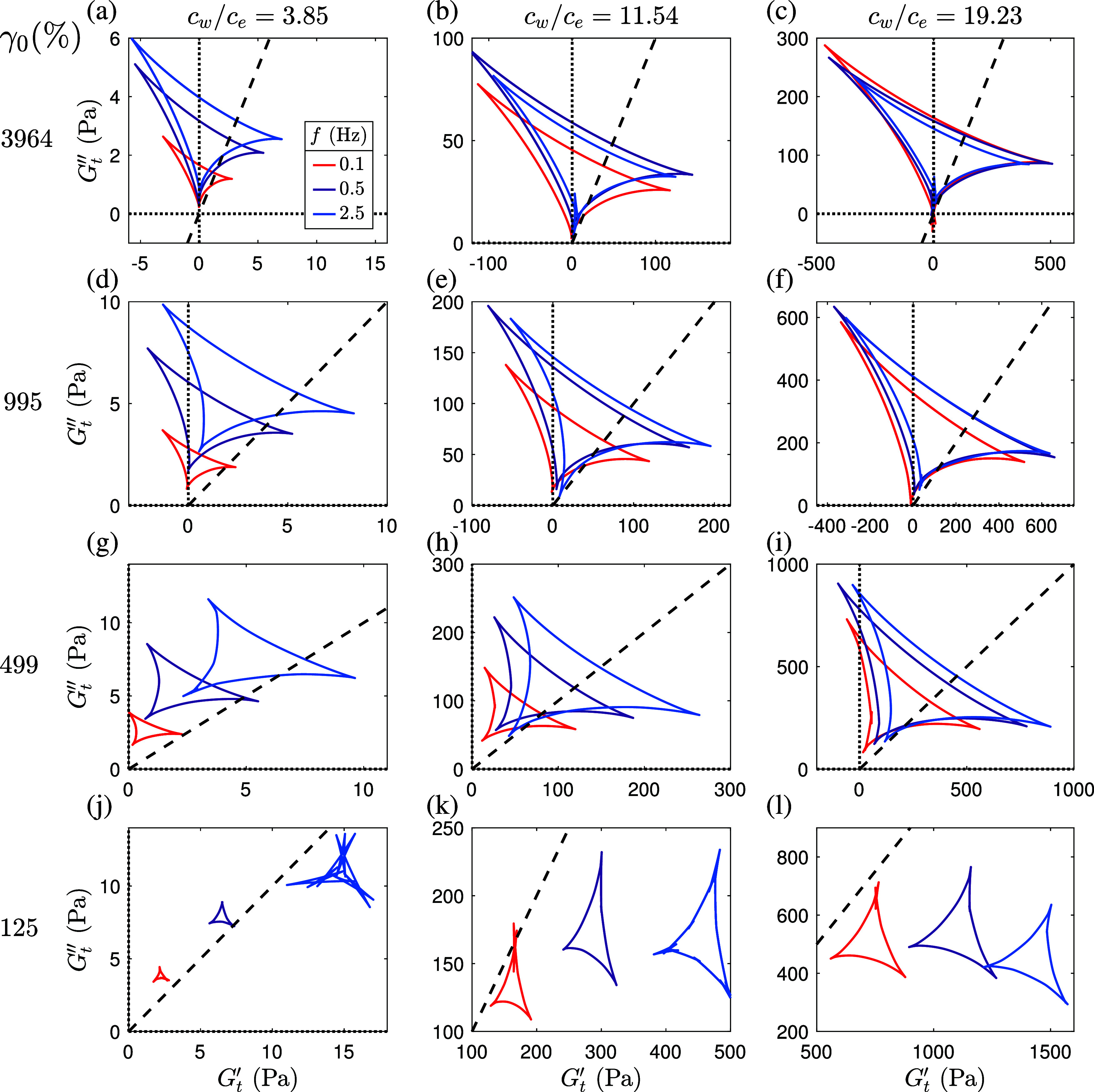
SPP analysis of the intracycle rheological transition at four strain amplitudes using Cole–Cole plot for PAAm concentrations of (a, d, g, j) *c*_*w*_/*c*_*e*_ = 3.85, (b, e, h, k) *c*_*w*_/*c*_*e*_ = 11.54 and (c, f, i, l) *c*_*w*_/*c*_*e*_ = 19.23, where (a–c) represent data for γ_0_ = 3964%, (d–f) represent data for γ_0_ = 995%, (g–i) represent data for γ_0_ = 499% and (j–l) represent data for γ_0_ = 125%. The dashed lines represent *G*_*t*_^′^ = *G*_*t*_^″^ and dotted lines represent *G*_*t*_^′^ = 0 and *G*_*t*_^″^ = 0.

#### 3.7.1 Key Stages of Polymer Structure Evolution During SPP

An example of the Cole–Cole plot for PAAm concentration of *c*_*w*_/*c*_*e*_ = 19.23 at γ_0_ = 995% and *f* = 0.5 Hz is shown in Figure 8 to understand the predicted intracycle rheological transitions within an oscillation cycle. In this particular case, the sequence of physical processes can be explained as sequence of *I: Initial Elastic Response (Stiffening)*. At the onset of deformation, the polymer network responds elastically on short timescales. This is characterized by an increase in *G*_*t*_^′^, corresponding to stiffening. The network structure, maintained by chain entanglements and hydrogen bonding, remains largely intact, and the material stores elastic energy. *II: Network Disruption and Thickening*. With increasing strain, physical cross-links (e.g., hydrogen bonds) are progressively disrupted, leading to a plastic-like flow (softening). However, the chains remain sufficiently entangled such that their reptation time scale is slower than the imposed deformation. As a result, the chains cannot fully relax, leading to local densification and an increase in *G*_*t*_^″^ (thickening). *III: Chain Relaxation and Thinning*. On longer timescales, the polymer chains are able to relax, resulting in a transition to bulk viscous flow. This is manifested as a decrease in *G*_*t*_^″^ (thinning), reflecting the increasing fluidization of the network as entanglements and associations are further disrupted. During the network disruption and chain relaxation stages, an *intracycle recoil* is observed, where *G*_*t*_^′^ becomes negative. This recoil likely reflects the recovery of residual elasticity from unbroken entanglements or the rebinding of hydrogen bonds, indicating a partial elastic recovery within the cycle.

These stages are robust across concentrations and frequencies (Figures 9 and S9), though their prominence and the location of the deltoids in the Cole–Cole plots shift with polymer concentration and oscillation frequency. At low concentrations (*c*_*w*_/*c*_*e*_ ≤ 3.85), the response is predominantly viscous with minimal elastic features, consistent with insufficient entanglement. At higher concentrations, the elastic feature becomes pronounced, and at large strains (γ_0_ ≳ 1000%), the intracycle transitions converge to a qualitatively similar sequence indicative of plastic-like flow behavior. This sequence provides a framework for interpreting the nonlinear viscoelasticity of entangled polymer solutions and highlights the critical role of network connectivity and dynamic associations in governing their rheological response. However, it is important to note that while the SPP framework provides insights into the intracycle transitions in a bulk-averaged sense, it does not reflect the spatial heterogeneity observed in rheomicroscopy. For example, at high strain amplitudes, rheomicroscopy reveals pronounced structural disruptions and localized fracture-like features in highly entangled samples (e.g., *c*_*w*_/*c*_*e*_ = 15.38), which are absent in less entangled systems like *c*_*w*_/*c*_*e*_ = 3.85 (Figure 7). This discrepancy highlights the limitation of bulk analyses in capturing localized failure modes, and motivates the integration of spatially resolved methods in future work.

#### 3.7.2 Quantifying Microstructural Rearrangement in an Oscillation Cycle

Other than locations, the deltoids area also changes significantly with increasing γ_0_ for different PAAm concentrations. At low concentrations, *c*_*w*_/*c*_*e*_ ≤ 3.85, the deltoids area (*A*_*d*_) increases with increasing γ_0_ up until very high strains, suggesting a continuous change to polymer solution structure during deformation^28,81^ (Figures 9 and 10a). At high concentrations, however, *c*_*w*_/*c*_*e*_ ≥ 11.54, there is a peak in the area of the deltoids (given by *A*_*d*,max_) at intermediate γ_0_, indicating that the microstructural rearrangement is highest at these strains (Figure 10a,b). *A*_*d*,max_ increases monotonically with PAAm concentrations suggesting a higher level of microstructural rearrangement within a cycle. This is likely a feature of increase in entanglement density and the energy required to remove topological constraints within the solution. The effect of the oscillation frequency on *A*_*d*,max_ is significant at low concentrations (*c*_*w*_/*c*_*e*_ = 3.85), where the *A*_*d*,max_ increases with increasing *f* (as also seen in the Cole–Cole plots in Figure 9a,d,g). At high concentrations, *A*_*d*,max_ values show a weak dependence on oscillation frequency (Figure 10b).

**Figure 10:**
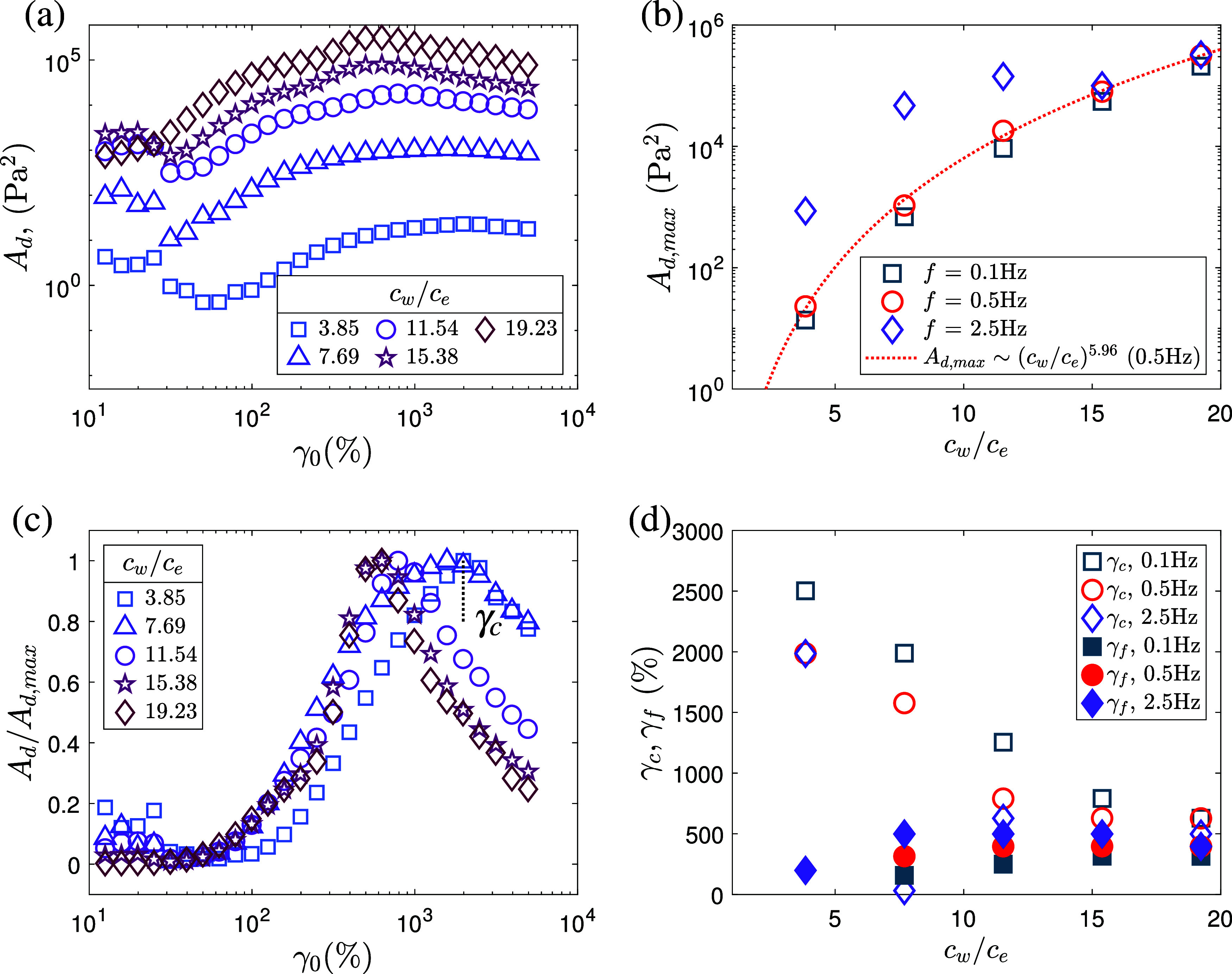
(a) Un-normalized deltoid area (*A*_*d*_) of all samples with respect to strain amplitudes at *f* = 0.5 Hz. (b) Maximum value of the deltoid areas (*A*_*d*,max_) as a function of PAAm concentrations. (c) Normalized deltoid area (*A*_*d*_/*A*_*d*,max_) of all samples with respect to strain amplitudes at *f* = 0.5 Hz. (d) Location of maximum value of the deltoid areas (γ_*c*_, shown in figure (c) for *c*_*w*_/*c*_*e*_ = 3.85 at *f* = 0.5 Hz) and the flow strain (γ_*f*_, calculated using the *G*′ = *G*″ crossover) as a function of PAAm concentrations for different oscillation frequencies, *f*. The results for *c*_*w*_/*c*_*e*_ = 1.54 is not shown because of high level of noise in the Cole–Cole plots.

The areas of the deltoids are normalized with their maximum values (*A*_*d*_/*A*_*d*,max_) to investigate the extent to which each sample recovers within a period with increasing strain amplitude. The critical strain (γ_*c*_) is the strain amplitude where the deltoids start to become smaller in size. Beyond γ_*c*_, the network can no longer rearrange itself and recover fast enough, evidenced by shrinkage in deltoid size.^28^ We propose that this corresponds to the strain in which greatest disentanglement, likely via convective constraint release, occurs.^82^ Past this strain, the flow becomes “fast” and topological entanglements decrease in number,^83^ and *A*_*d*_/*A*_*d*,*max*_ decreases rapidly. The location of *A*_*d*_/*A*_*d*,max_ is at the highest γ_0_ (γ_0_ ≈ 2000%) for the lowest PAAm concentration, *c*_*w*_/*c*_*e*_ = 3.85 (Figure 10c). This suggests that the microstructural rearrangement for *c*_*w*_/*c*_*e*_ = 3.85 is maximum at a higher strain compared to materials with higher PAAm concentrations. It should be noted here that *A*_*d*_/*A*_*d*,max_ is a relative quantity, and the microstructural rearrangement and therefore the *A*_*d*,max_ remains higher for the sample with higher PAAm concentrations. For *c*_*w*_/*c*_*e*_ ≥ 15.38, the locations of *A*_*d*_/*A*_*d*,max_ become very similar to each other, at around γ_0_ ≈ 600%.

A comparison between the end of flow transition region (or the onset of bulk flow region) determined by the crossover of *G*′ and *G*″ (denoted as γ_*f*_), and the critical strain values where the normalized deltoid size increases to its maximum value (denoted as γ_*c*_) is made. γ_*c*_ > γ_*f*_ for all the PAAm concentrations, where the difference is highest at the lowest PAAm concentration (Figure 10). For example, at *c*_*w*_/*c*_*e*_ = 3.85 and *f* = 2.5 Hz, γ_*c*_ ≈ 2000% and γ_*f*_ ≈ 200% (around 10 times higher). Despite the moduli crossover occurring at γ_*f*_ ≈ 200%, the sample’s microstructural rearrangement is maximum at around γ_*c*_ ≈ 2000%. This difference decreases with increasing PAAm concentrations and becomes almost constant for *c*_*w*_ ≳ 10wt%, where γ_*c*_ is around 1.4 times higher than γ_*f*_ at *f* = 0.5 Hz. There also exists a dependence of γ_*c*_ and γ_*f*_ on *f*. The lower the *f*, the larger is the difference between these γ_*c*_ and γ_*f*_ values (Figure 10d). This suggests that the moduli crossover, which is commonly associated with the onset of bulk flow under LAOS, including energy dissipation ratio (ϕ), intrinsic nonlinearity (^3^ *Q*_0_), and intracycle transitions, indicate that semidilute entangled PAAm solutions undergo yielding and plastic-like flow under large amplitude deformation.

In summary, the Cole–Cole analysis reveals that polymer concentration significantly alters intracycle rheological transitions in the linear and transition regimes. However, once the solutions enter the flow regime, the SPP-derived trajectories show a convergence toward plastic-like behavior across all concentrations. Rheomicroscopy, however, provides more detailed picture: while low-concentration samples deform more homogeneously across all strains, high-concentration samples display distinct flow heterogeneities, including banding and fracture-like discontinuities. These spatial features are not captured by SPP or other bulk rheological measures such as ϕ or *I*_3/1_, which reflect only spatially averaged behavior. The critical strain γ_*c*_, identified from normalized deltoid area, offers a useful metric for quantifying microstructural disruption, but should be interpreted in conjunction with direct imaging data to avoid overgeneralizing about flow uniformity.

## 4 Conclusions

This study provides a comprehensive rheological investigation of aqueous polyacrylamide (PAAm) solutions across a broad concentration range (0.1–12.5 wt%), normalized by the entanglement concentration *c*_*e*_ = 0.65 wt%. By presenting our results as a function of the normalized concentration *c*_*w*_/*c*_*e*_ (ranging from 0.15 to 19.23), a unified comparison of mechanical behavior across regimes from semidilute unentangled to highly entangled solutions is enabled. With increasing *c*_*w*_/*c*_*e*_, the solutions transition from viscous-dominated liquids (*c*_*w*_/*c*_*e*_ < 1) to elastic-dominated fluids (*c*_*w*_/*c*_*e*_ > 3), exhibiting strong nonlinear viscoelasticity and pseudoplastic behavior. While these systems display yield stress-like features under large amplitude oscillatory shear (LAOS), steady shear tests suggest that they do not possess a true yield stress, even at the highest concentrations (*c*_*w*_/*c*_*e*_ ∼ 19). Instead, this is interpreted as an operational transition between reversible, elastic deformation to irreversible microstructural rearrangement. SAOS measurements reveal a concentration-dependent crossover from *G*″ > *G*′ to *G*′ > *G*″, typically between *c*_*w*_/*c*_*e*_ ≈ 1.5–3.85, marking the onset of elastic network formation. Frequency dependence of moduli diminishes at high *c*_*w*_/*c*_*e*_, consistent with slower structural relaxation and transient entanglements. LAOS analyses identify three key regimes: linear, transition, and bulk flow, quantified via harmonic ratios, intrinsic nonlinearity (^3^ *Q*_0_), and energy dissipation ratio (ϕ). Notably, ϕ decreases in the linear regime with increasing *c*_*w*_/*c*_*e*_, and converges to ∼0.88 at large strain amplitudes across all samples, indicating a similar plastic-like dissipation mechanism despite different microstructures.

The sequence of physical processes (SPP) framework reveals robust intracycle transitions: stiffening, thickening, relaxation, and recoil, that persist across *c*_*w*_/*c*_*e*_, though their magnitude and symmetry depend on entanglement density and deformation rate. However, bulk rheology alone does not provide critical information about the spatio-temporal features the samples undergo during large deformation. Rheomicroscopy observations fill this gap, revealing concentration-dependent flow heterogeneity. At low concentrations (*c*_*w*_/*c*_*e*_ ≲ 3.85), samples remain optically homogeneous even under high strain. In contrast, high concentration solutions (*c*_*w*_/*c*_*e*_ ≳ 15) show clear signs of structural disruption, including shear banding and fracture-like features. These observations emphasize the importance of coupling bulk rheology with spatially resolved techniques such as rheo-microscopy, as traditional bulk metrics may obscure local flow instabilities.

This study demonstrates the significance of integrating normalized concentration scaling (*c*_*w*_/*c*_*e*_), nonlinear viscoelastic metrics (e.g., ϕ, ^3^ *Q*_0_, SPP), and real-time imaging to uncover the complex flow transition behavior of polymer solutions. Our findings offer new mechanistic insight into transiently entangled fluids and establish a foundation for predictive rheology in soft material formulation and processing.

## Supplementary Material







## Notes

Rheomicroscopy PAAm cwbyce 3p85 (AVI)
Rheomicroscopy PAAm cwbyce 15p38 (AVI)
GPC of PAAm (Figure S1); protorheology test at the highest PAAm concentration (Figure S2); storage and loss moduli (Figure S3); comparison of steady shear viscosity measured using steady-state sensing times of 20 s and 60 s (Figure S4); steady shear stress versus shear rate (Figure S5); elastic Lissajous–Bowditch curves (Figure S6); viscous Lissajous–Bowditch curves (Figure S7); FT spectra of stress response (Figure S8); and SPP Cole–Cole plots (Figure S9) (PDF)
