# Supplementary material for: Nonlinear Rheological Behavior of Polyacrylamide Solutions under Large-Amplitude Oscillatory Shear
Source: Macromolecules. 2026 Mar 31;59(7):4084–100. doi: 10.1021/acs.macromol.5c02848 (PMC13085858; doi:10.1021/acs.macromol.5c02848)
Supplement: Supplementary file 3 [file ma5c02848_si_003.pdf]

Nonlinear rheological behaviour of polyacrylamide solutions under large amplitude oscillatory shear

Rishav Agrawal,*,†,‡ William N. Sharratt,† Robert J. Poole,† and Esther García-Tuñón†,‡

†School of Engineering, University of Liverpool, The Quadrangle, L69 3GH Liverpool, United Kingdom

‡Materials Innovation Factory, University of Liverpool, 51 Oxford Street, Liverpool L7 3NY, United Kingdom

E-mail: rishavagr@gmail.com

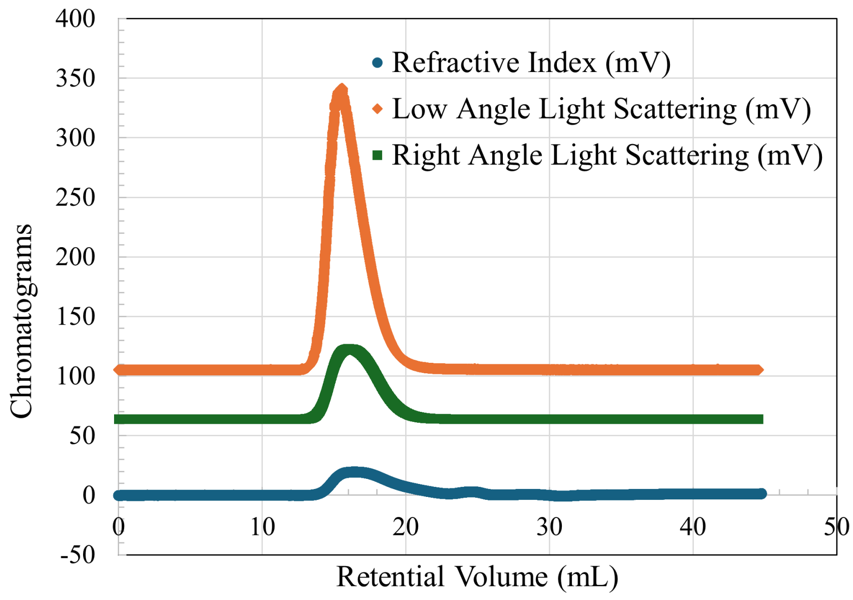

Figure S1: Chromatograms of PAAm ran under 0.2M $NaNO_3$ + 0.01M NaH_2PO_4 at pH 7 (aq).

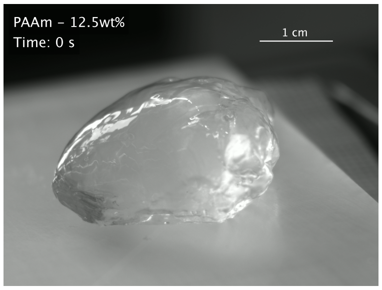

(a) t = 0s

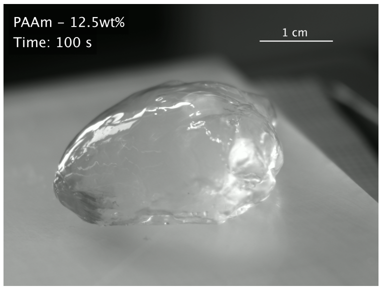

(b) t = 100s

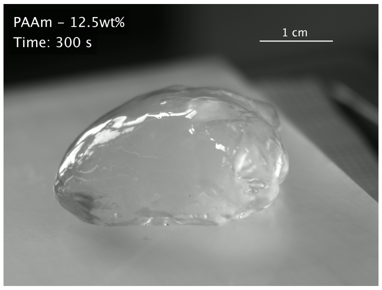

(c) t = 300s

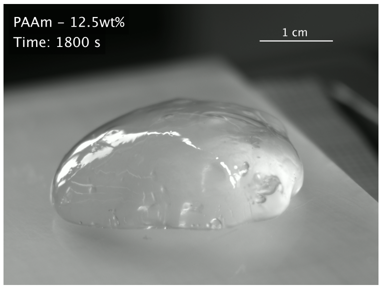

(d) t = 1800s

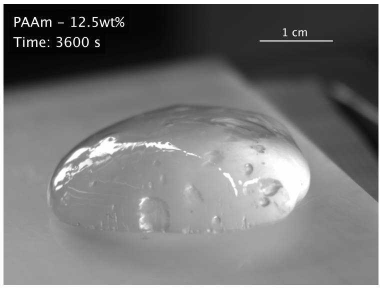

(e) t = 3600s

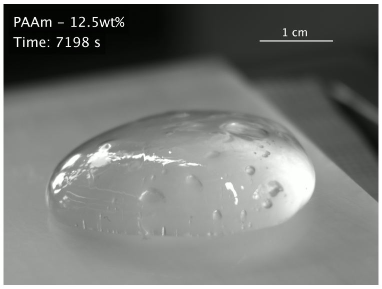

(f) t = 7199s

Figure S2: Protorheology test on the highest-concentration PAAm solution (c_w/c_e = 19.23). Panels (a)–(f) show the sample at successive time stamps following placement on a flat PTFE surface at room temperature (22 $^{\circ}$C). Despite its initially solid-like appearance, the sample gradually deforms under gravity over a 2-hour period, indicating that any yield stress present is weak and insufficient to prevent flow under its own weight. This qualitative observation is consistent with the steady shear results and supports the interpretation that the solution behaves as a strongly viscoelastic, shear-thinning fluid rather than a material with a well-defined yield stress.

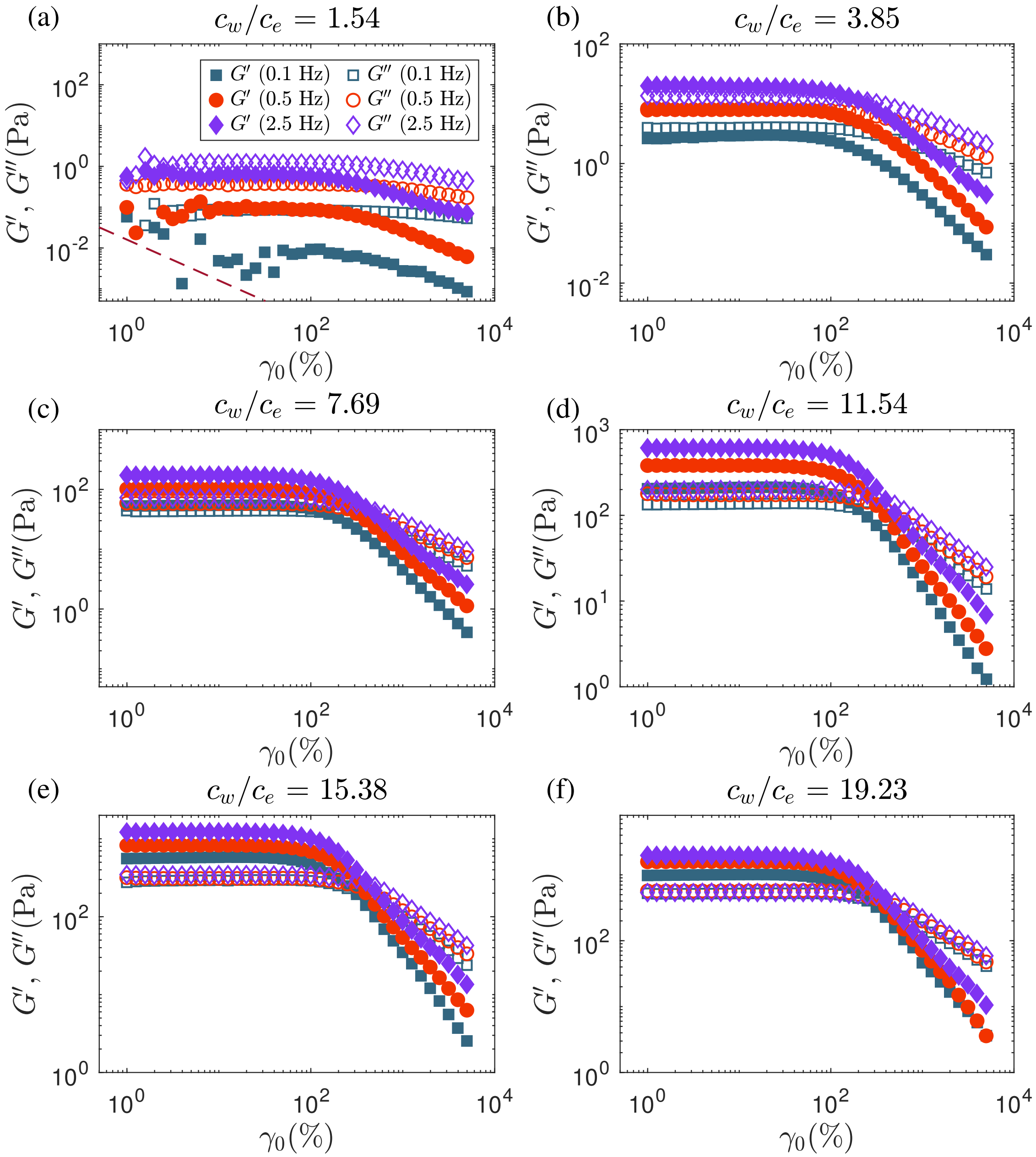

Figure S3: Variation of G' and G'' with increasing strain amplitudes at various oscillation frequencies for (a) $c_w/c_e = 1.54$, (b) $c_w/c_e = 3.85$, (c) $c_w/c_e = 7.69$, (d) $c_w/c_e = 11.54$, (e) $c_w/c_e = 15.38$, and (f) $c_w = 19.23$. The red dashed line in (a) represents the minimum measurable shear stress, $\sigma_{min,osc} \approx 0.016$ Pa of the ARES G2 rheometer, based on the manufacturer-specified minimum torque, $T_{min,osc} = 0.05\mu$N.m.

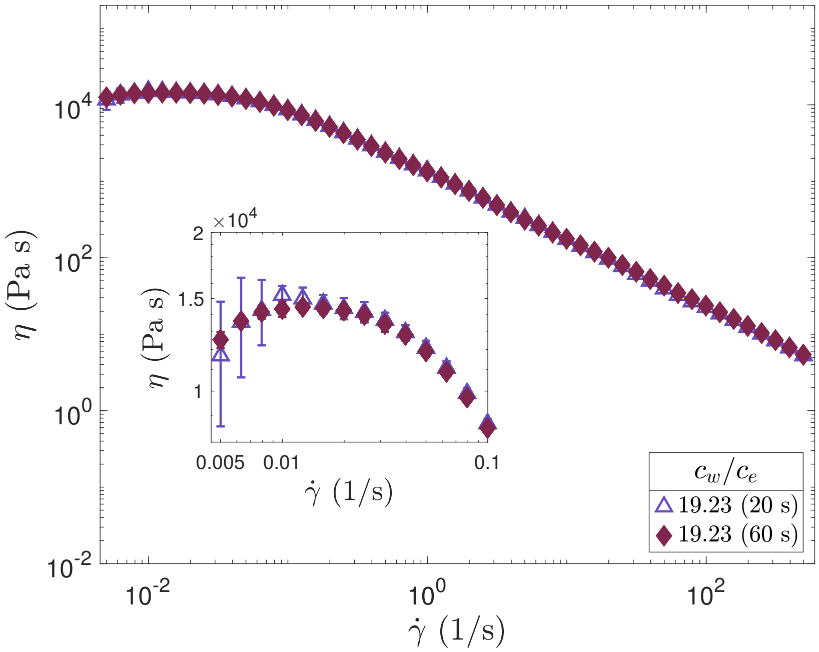

Figure S4: Comparison of steady-shear viscosity for the PAAm solution at $c_w/c_e = 19.23$, measured using steady-state sensing times of 20s and 60s per shear-rate step. The viscosity curves overlap almost over the full shear-rate range, while the 20s measurements exhibit larger scatter and uncertainty at the lowest shear rates (inset). Extending the sensing time to 60s reduces this variability without altering the overall shear-thinning behaviour.

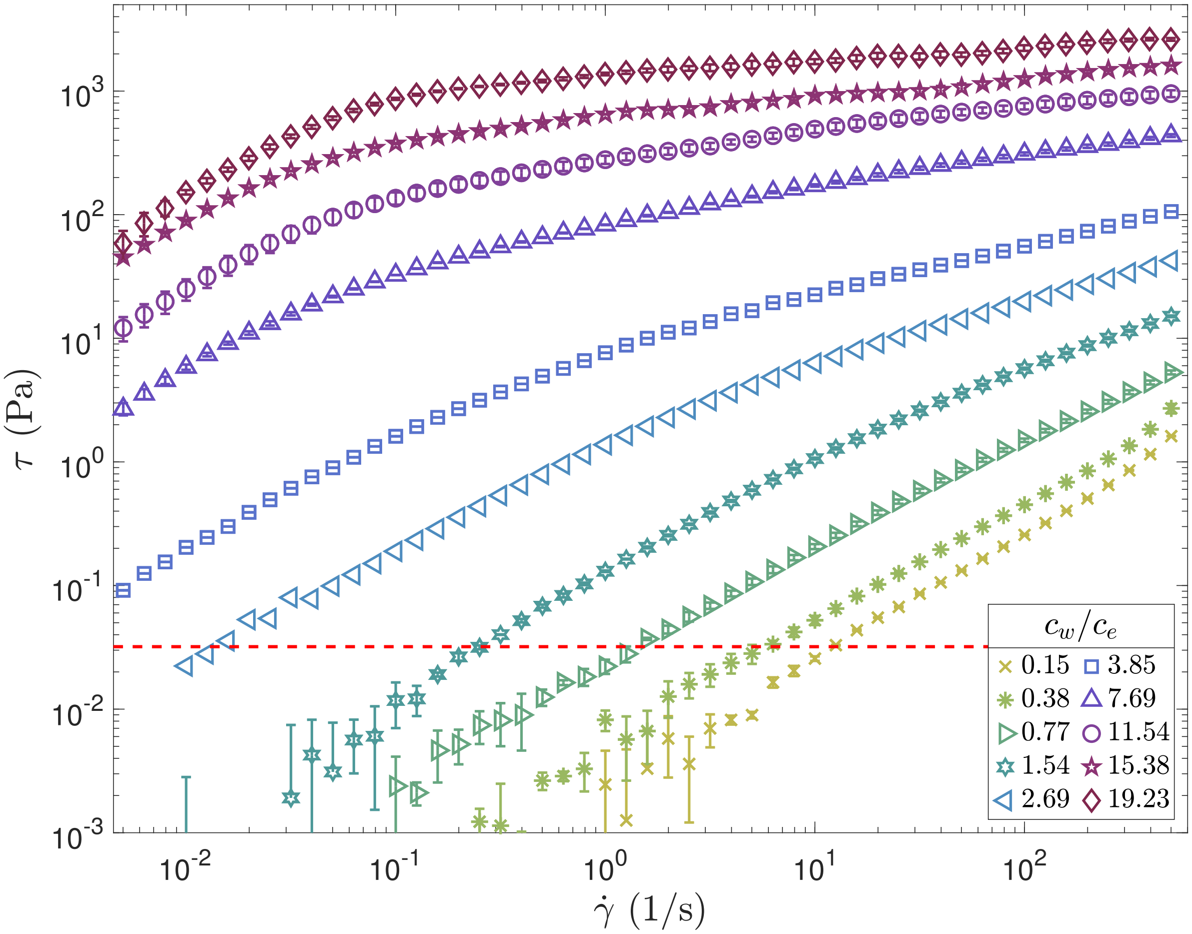

Figure S5: Steady shear stress-shear rate profiles for PAAm concentrations ranging from c_w/c_e = 0.15 - 19.23. The red dashed line denotes the minimum measurable shear stress, $\sigma_{\mathrm{min,ss}} \approx 0.032$ Pa, for the ARES G2 rheometer, based on the manufacturer-specified minimum torque $T_{\mathrm{min,ss}}$ = 0.1μN·m.

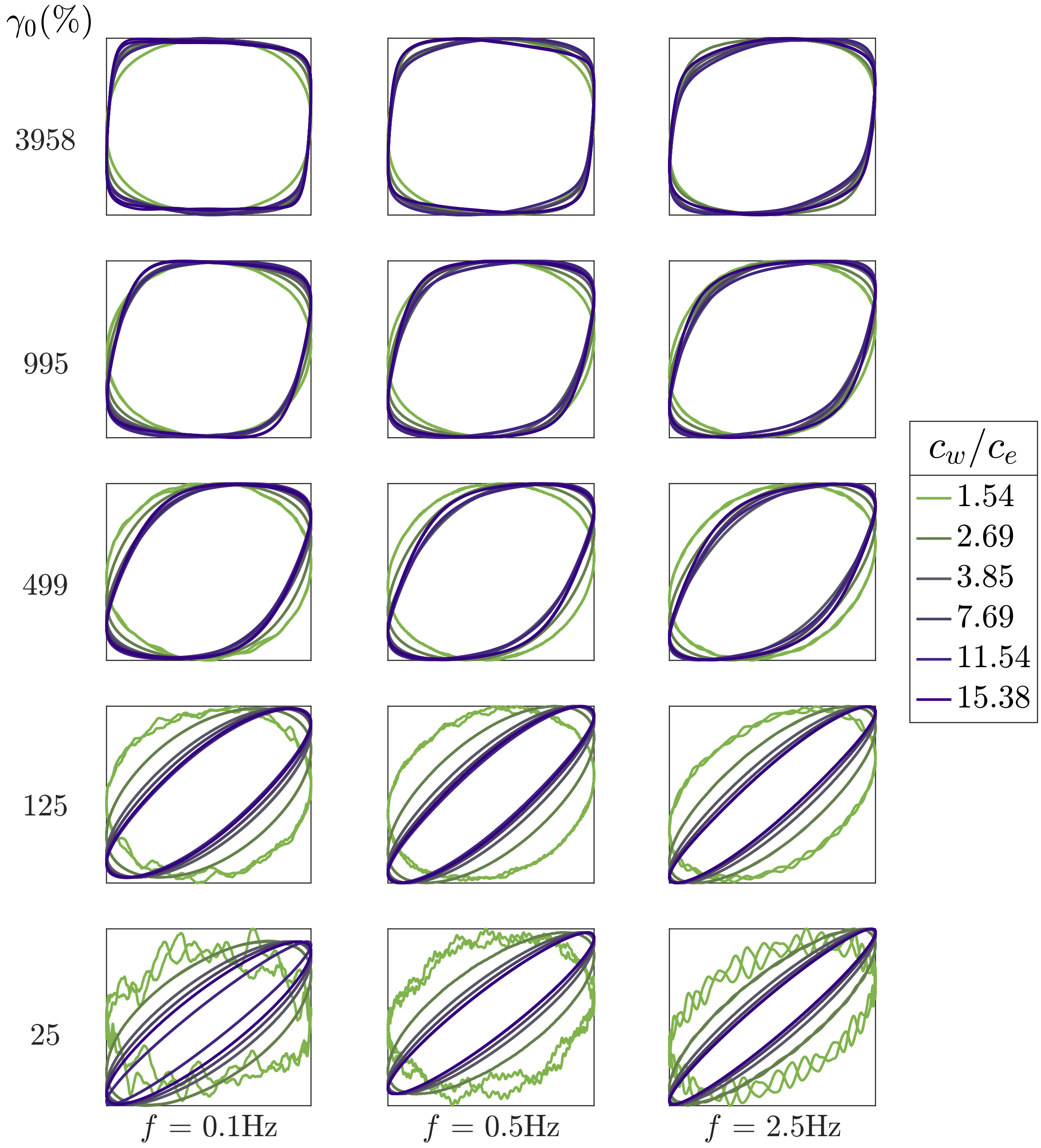

Figure S6: Normalized elastic Lissajous-Bowditch (LB) curves (σ/σ_0 vs γ/γ_0) of PAAm solutions at different concentrations for three oscillation frequencies (f = 0.1Hz, 0.5Hz, and 2.5Hz, left to right). Strain amplitudes increase from bottom to top. We note here that the data for c_w/c_e = 1.54 at low γ_0 remain close to the instrument resolution.

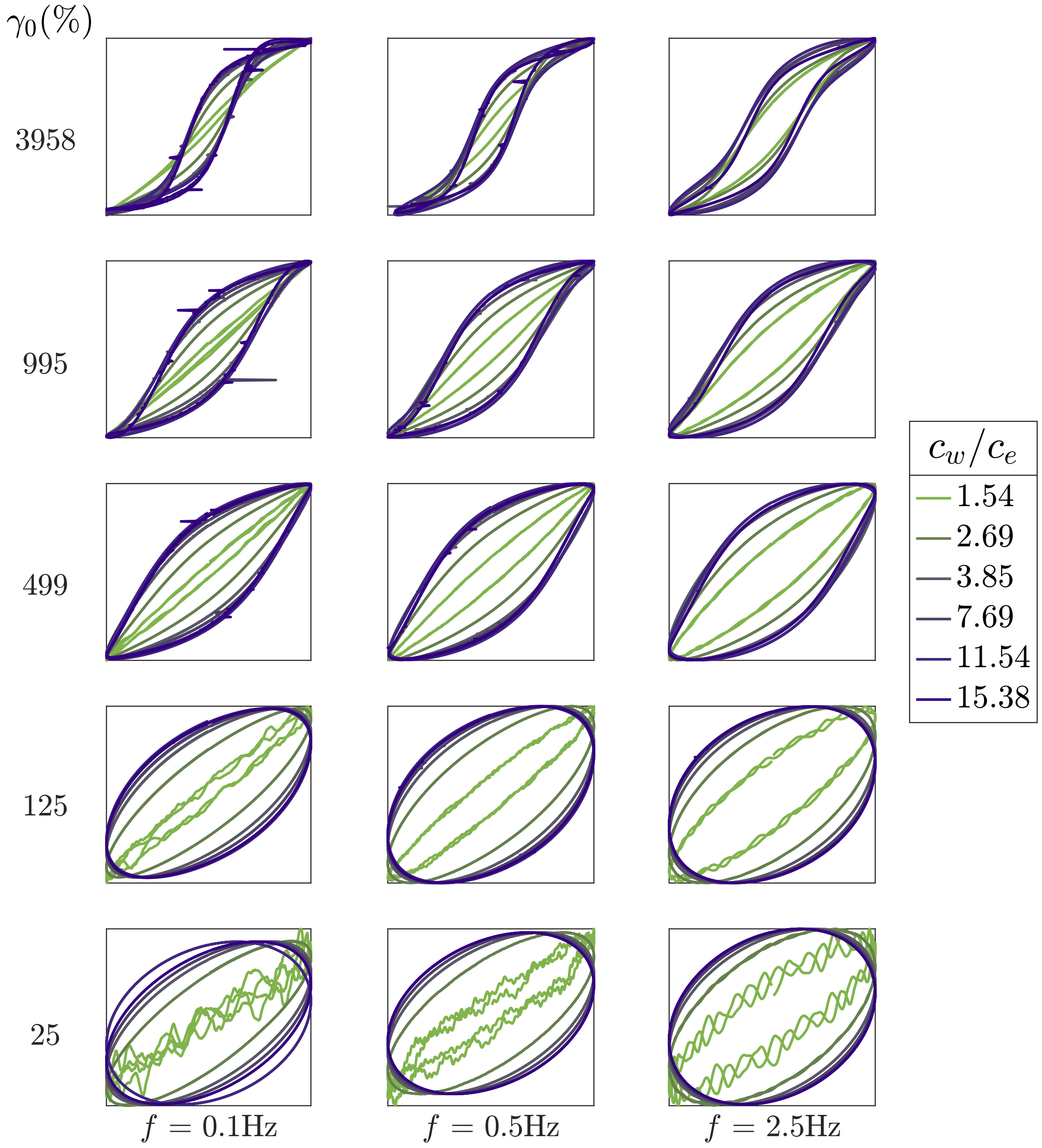

Figure S7: Normalized viscous Lissajous-Bowditch (LB) curves (σ/σ_0 vs $\dot{\gamma}/\dot{\gamma}_0$) of PAAm solutions at different concentrations for three oscillation frequencies (f = 0.1Hz, 0.5Hz, and 2.5Hz, left to right). Strain amplitudes increase from bottom to top. We note here that the data for c_w/c_e = 1.54 at low γ_0 remain close to the instrument resolution.

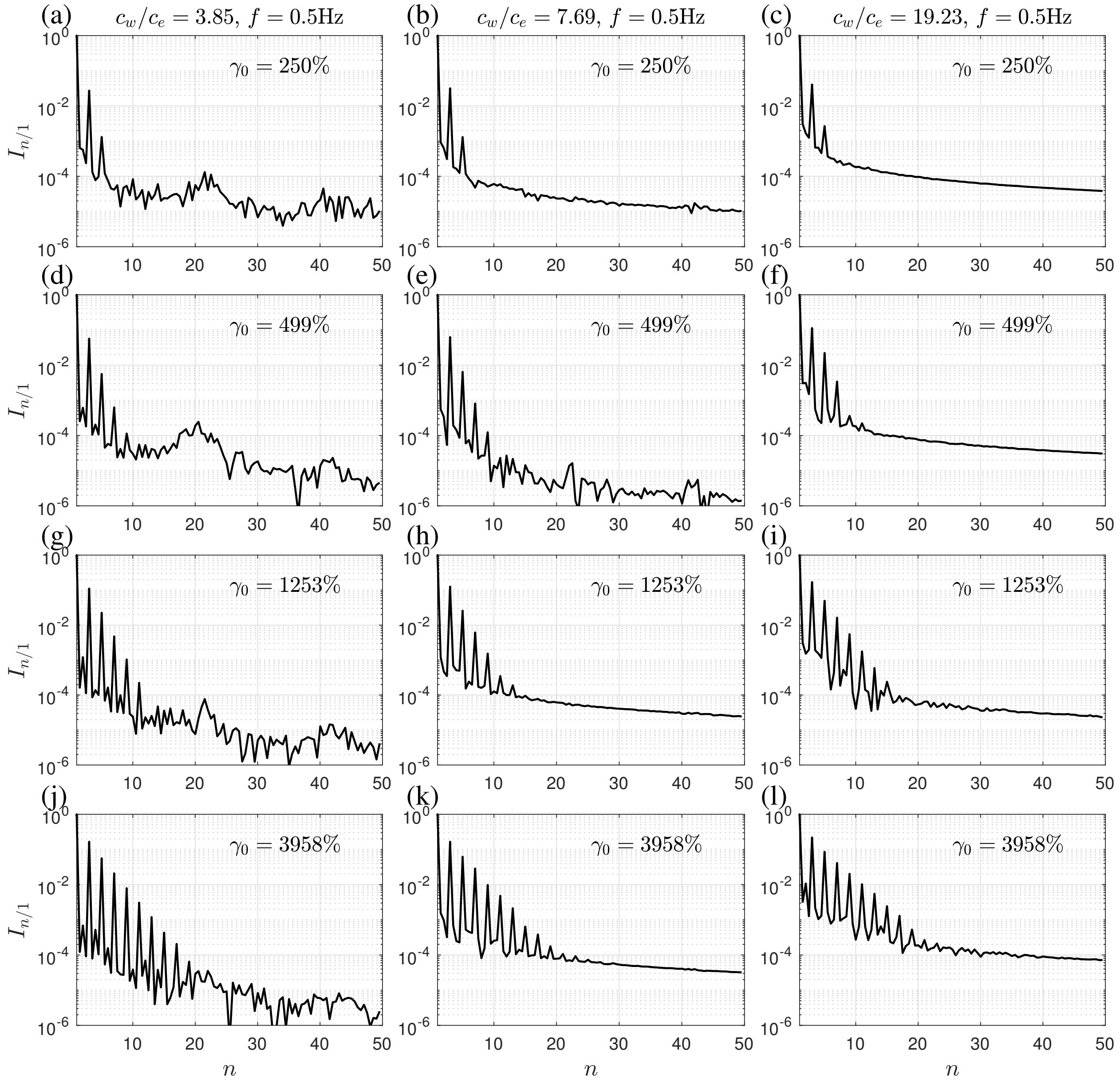

Figure S8: Representative Fourier-transform (FT) spectra of the stress response for PAAm solutions at $f = 0.5$Hz, shown for three concentrations ($c_w/c_e = 3.85, 7.69$ and 19.23) and selected strain amplitudes. The spectra display the normalized harmonic intensities $I_{n/1}$ as a function of harmonic number n, illustrating the raw spectral data underlying the calculation of $I_{3/1}$, Q_0, and SPP metrics, as well as the practical noise floor achieved in the experiments.

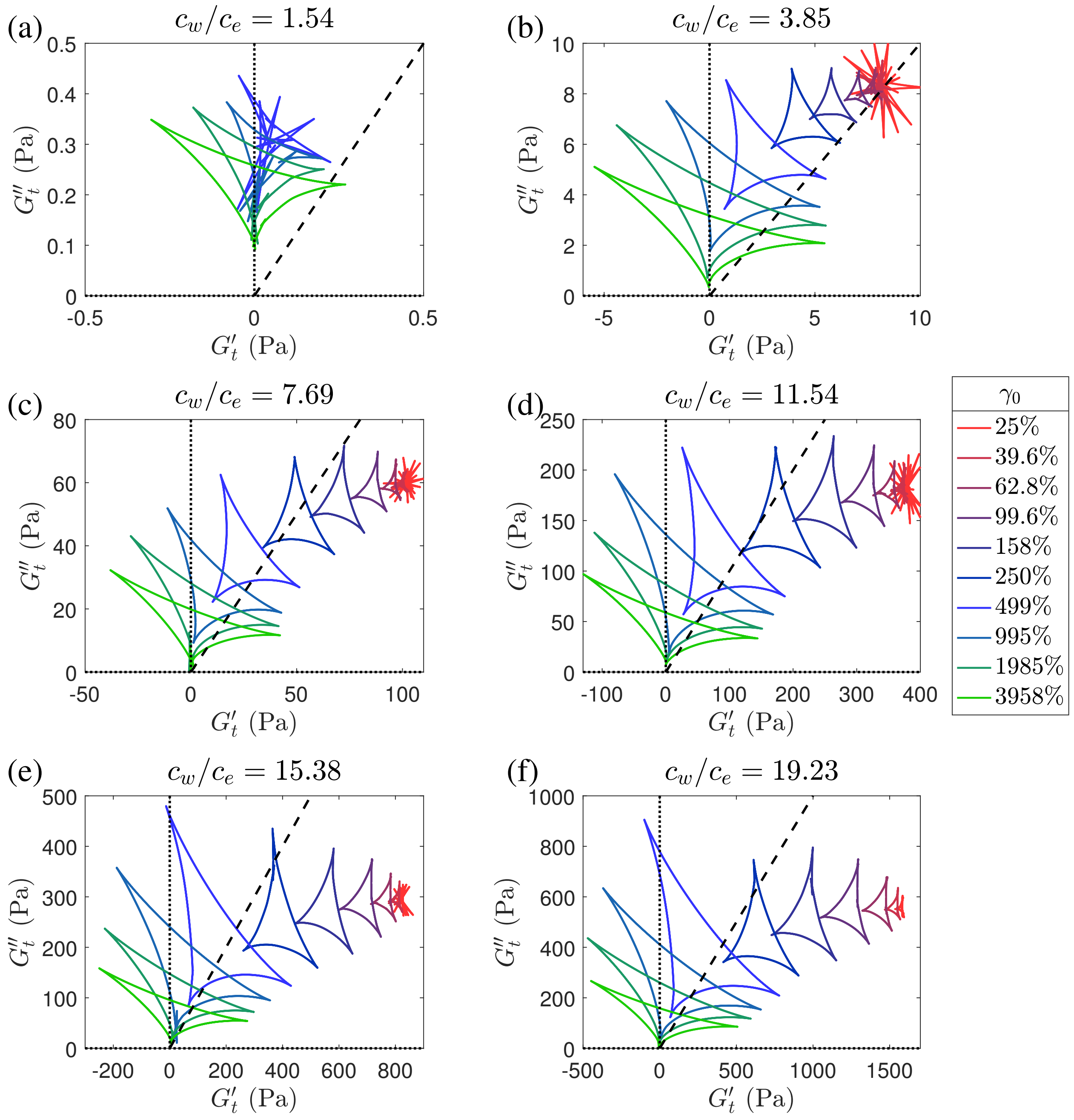

Figure S9: SPP analysis of the intra-cycle rheological transition at $f = 0.5$Hz using Cole–Cole plot for PAAm concentrations of c_w/c_e = (a) 1.54, (b) 3.85, (c) 7.69, (d) 11.54, (e) 15.38 and (f) 19.23. The dashed and dotted lines represent $G'_t = G''_t$ and $G'_t = 0$, respectively. The low γ_0 data for c_w/c_e = 1.54, is not shown due to a high level of noise.
